# Phylogeny, body morphology, and trophic level shape intestinal traits in coral reef fishes

**DOI:** 10.1002/ece3.8045

**Published:** 2021-08-29

**Authors:** Mattia Ghilardi, Nina M. D. Schiettekatte, Jordan M. Casey, Simon J. Brandl, Samuel Degregori, Alexandre Mercière, Fabien Morat, Yves Letourneur, Sonia Bejarano, Valeriano Parravicini

**Affiliations:** ^1^ Reef Systems Research Group Department of Ecology Leibniz Centre for Tropical Marine Research (ZMT) Bremen Germany; ^2^ Department of Marine Ecology Faculty of Biology and Chemistry University of Bremen Bremen Germany; ^3^ PSL Université Paris: EPHE‐UPVD‐CNRS USR3278 CRIOBE Perpignan France; ^4^ Laboratoire d’Excellence “CORAIL” Perpignan France; ^5^ Department of Marine Science Marine Science Institute University of Texas at Austin Port Aransas TX USA; ^6^ CESAB Centre for the Synthesis and Analysis of Biodiversity Institut Bouisson Bertrand Montpellier France; ^7^ Department of Ecology and Evolutionary Biology University of California Los Angeles Los Angeles CA USA; ^8^ UMR ENTROPIE (UR‐IRD‐CNRS‐IFREMER‐UNC) Université de la Nouvelle‐Calédonie Nouméa Cedex New Caledonia; ^9^ Institut Universitaire de France Paris France

**Keywords:** Bayesian phylogenetic comparative method, convergent evolution, digestive traits, evolutionary conservatism, fish diet, gut length

## Abstract

1. Trait‐based approaches are increasingly used to study species assemblages and understand ecosystem functioning. The strength of these approaches lies in the appropriate choice of functional traits that relate to the functions of interest. However, trait–function relationships are often supported by weak empirical evidence.
2. Processes related to digestion and nutrient assimilation are particularly challenging to integrate into trait‐based approaches. In fishes, intestinal length is commonly used to describe these functions. Although there is broad consensus concerning the relationship between fish intestinal length and diet, evolutionary and environmental forces have shaped a diversity of intestinal morphologies that is not captured by length alone.
3. Focusing on coral reef fishes, we investigate how evolutionary history and ecology shape intestinal morphology. Using a large dataset encompassing 142 species across 31 families collected in French Polynesia, we test how phylogeny, body morphology, and diet relate to three intestinal morphological traits: intestinal length, diameter, and surface area.
4. We demonstrate that phylogeny, body morphology, and trophic level explain most of the interspecific variability in fish intestinal morphology. Despite the high degree of phylogenetic conservatism, taxonomically unrelated herbivorous fishes exhibit similar intestinal morphology due to adaptive convergent evolution. Furthermore, we show that stomachless, durophagous species have the widest intestines to compensate for the lack of a stomach and allow passage of relatively large undigested food particles.
5. Rather than traditionally applied metrics of intestinal length, intestinal surface area may be the most appropriate trait to characterize intestinal morphology in functional studies.

## Graphical abstract

Intestinal morphology is key to integrating digestive and assimilative processes into trait‐based approaches. Yet intestinal length alone has been commonly use for this purpose in fishes. Our study demonstrates that evolution and ecology have shaped a great diversity of intestinal morphologies in coral reef fishes that cannot be captured by length alone. We show that intestinal traits are clear indicators of fish trophic roles and intestinal surface area may be a most suitable trait to apply in functional studies than intestinal length.

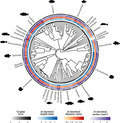

## 1 INTRODUCTION

Characterizing the relationship between form and function provides information on the evolutionary history of species, their potential to adapt to new environmental conditions, and their role within ecosystems. Form and function are often closely related (Wainwright, 1988), as is evident across a wide variety of taxonomic groups, such as invertebrates (Griffen & Mosblack, 2011; Wang et al., 1997) and large mammals (Ekdale, 2016; Hutchinson et al., 2011). However, determining whether the relationship between form and function is driven by evolutionary processes (Banavar et al., 2014; Westneat, 1995) or environmental conditions (Herrel et al., 2008; Naya et al., 2014) remains difficult to pinpoint.

Intestinal morphology is central to one of the most important organismal processes—the digestion of prey sourced from the environment—and as such likely to have tight links to functional roles. Indeed, characteristics of the intestine and other digestive organs are associated with energy assimilation (Battley & Piersma, 2005; Cleveland & Montgomery, 2003) and thus the persistence of populations (Brewster et al., 2020). Further, intestinal morphology is strongly related to diet in both vertebrate and invertebrate groups (Griffen & Mosblack, 2011; Steinberg, 2018). For instance, intestinal length is negatively correlated with trophic level in mammals (Korn, 1992; Wang et al., 2003), birds (Al‐Dabbagh et al., 1987; Battley & Piersma, 2005; Ricklefs, 1996), reptiles (O’Grady et al., 2005), amphibians (Naya et al., 2009), and fishes (Kramer & Bryant, 1995b; Elliott & Bellwood, 2003; reviewed in Steinberg, 2018). Primary consumers generally require long intestines because they need to acquire energy and nutrients from plants with low nutritional value and high fiber content (Horn, 1989). However, building and maintaining a long intestine has high evolutionary and physiological costs (Cant et al., 1996). Intestinal morphology therefore represents a trade‐off between the benefits of nutrient acquisition and the costs of maintaining a large organ.

Beyond diet, evolutionary processes also play a role in shaping intestinal morphology (Lauder, 1981). Phylogenetic conservatism has been identified across several taxa (Davis et al., 2013; German et al., 2010; Hunt et al., 2019), suggesting that evolution can constrain intestinal morphological variation within certain size ranges. However, species can overcome phylogenetic conservatism through phenotypic flexibility, which allows organisms to adapt to local environmental conditions (Piersma & Lindström, 1997). For example, some vertebrates can respond to changing environmental conditions by adjusting the structure and physiology of their gastro‐intestinal tracts (Battley & Piersma, 2005; Dala‐Corte et al., 2017; Herrel et al., 2008; Starck, 2003). Intestinal structural flexibility has been observed in response to fasting (Starck & Beese, 2002; Zaldúa & Naya, 2014), increased food intake (Dykstra & Karasov, 1992; Starck & Beese, 2001), changes in diet (Naya et al., 2007; Olsson et al., 2007), and through ontogenetic development (Kramer & Bryant, 1995a).

Coral reefs host an extraordinary diversity of species. Among these species, fishes are the most diverse and prominent vertebrates, exhibiting a wide array of morphologies and trophic strategies (Alfaro et al., 2007; Cowman et al., 2009; Floeter et al., 2018; Parravicini et al., 2020; Price et al., 2011, 2013; Siqueira et al., 2020). Given this multitude of feeding behaviors, reef fishes represent an ideal group to study how evolutionary and ecological mechanisms influence intestinal morphology.

Reef fish intestinal morphology has been related to the quality of their diet (Al‐Hussaini, 1947; Elliott & Bellwood, 2003; Emery, 1973). However, several limitations have hampered a full understanding of the nature and strength of this relationship. First, previous studies are often limited to single taxonomic families (Berumen et al., 2011; Wagner et al., 2009). Second, most studies focus on intestinal length, which, alone, does not fully describe intestinal morphology (Elliott & Bellwood, 2003). Third, evolutionary constraints on intestinal morphology have only been considered across a limited number of taxonomic groups (Davis et al., 2013; Wagner et al., 2009). Fourth, while intestinal traits have always been corrected for allometry, no study has accounted for body shape. Lastly, other digestive organs, such as the stomach, may impact this relationship, but this has never been investigated. Thus, a better understanding of the digestive traits and trophic roles of reef fishes may come from a broader, more diverse, and multifaceted assessment of digestive traits in reef fishes.

Here, we assess the main drivers of variability in the intestinal morphology of coral reef fishes. We investigate differences in intestinal length, diameter, and surface area of 1,208 individuals belonging to 142 species and 31 families collected in Mo'orea, French Polynesia. Specifically, we use Bayesian phylogenetic hierarchical analysis to disentangle the relationship among intestinal morphological traits and phylogeny, body size, body shape, diet, and the presence of the stomach. Further, we investigate the body size relationship at both the inter‐ and intraspecific level.

## 2 MATERIALS AND METHODS

### 2.1 Data collection

A total of 1,208 individuals from 142 species were collected from reefs around Mo'orea, French Polynesia, in the lagoon, pass, and outer reef slope (Appendix S2: Table 1), between 2018 and 2019. We primarily targeted adult fishes, but a wider size range was collected for a subset of species. The selection of species cover all the major trophic guilds of coral reef fishes (i.e., corallivores, herbivores, invertivores, piscivores, and planktivores). All individuals were collected by spearfishing between 10:00 and 15:00 hours and transferred to the laboratory on ice. In the laboratory, each individual was measured and weighed, and the intestine was unraveled and photographed on a tray, using a ruler for a size reference. A minimum of three individuals per species was examined. The collection of fishes for this project was approved by the Ministry of the Environment of French Polynesia (permit #681/MCE/ENV).

We measured the length and the external diameter of the intestine using the software Fiji/ImageJ (Schindelin et al., 2012). The length was measured from the pyloric outlet to the anus in the presence of a stomach and from the esophagus to the anus in stomachless fishes (Elliott & Bellwood, 2003; Karachle & Stergiou, 2010; Kramer & Bryant, 1995a, 1995b). The average diameter was calculated with measurements taken at ten equal intervals along the entire length of the intestine (Elliott & Bellwood, 2003). The external intestinal surface area (IS) was used as a proxy for mucosal surface area (Cleveland & Montgomery, 2003; Lassuy, 1984; Montgomery, 1977), and it was estimated using the following formula:

(1) IS=2πr×IL,

where *r* is the mean outer radius of the intestine, and IL is the intestinal length. Notably, scraping and excavating species of parrotfishes (genera *Chlorurus* and *Scarus*, *n* = 10 species) have ileal sacculations (Clements & Choat, 2018), leading to a potential underestimation of their intestinal surface area by this formula; yet, all other species examined in this study have a smooth external intestinal surface, suggesting accurate quantifications via the applied formula. Thus, while our calculation is a coarse estimation of mucosal surface area that does not account for mucosal folding, it can be considered a valid indicator of general intestinal surface area across most species (Cleveland & Montgomery, 2003; Lassuy, 1984; Montgomery, 1977).

Each species was classified based on the presence or absence of a functional stomach. We considered both gastric and muscular (gizzard‐like, *n* = 5) stomachs to be functional stomachs because they contribute to food digestion. In contrast, sac‐like stomachs (e.g., Tetraodontidae) were considered nonfunctional stomachs. Furthermore, species were classified as either durophagous or not durophagous depending on whether their diet consisted of hard‐shelled prey items (e.g., corals, crabs, molluscs, and sea urchins). We compiled this dataset according to the literature (Fagundes et al., 2016; Koide & Sakai, 2021; Ray & Ringø, 2014; Sorenson et al., 2013; Wilson & Castro, 2010), authors’ knowledge, and direct observation of the dissected fishes.

We used trophic level as a continuous measure of diet. Data were retrieved from FishBase using the R package *rfishbase* version 3.0.4 (Boettiger et al., 2012). In FishBase, a species’ trophic level is calculated by adding one to the mean trophic level of all food items consumed, weighted by their contribution (Froese & Pauly, 2000). Two estimates of trophic level are available: one based on diet composition and the other based on food items. The diet‐based index is only available for a few of our species, so the food item‐based index was used as a measure of trophic level and, when unavailable, the mean value of the genus (*n* = 14) or family level (*n* = 1) was used.

Since food item‐based trophic levels reflect temporal snapshots of gut contents, they may not represent a species’ entire dietary breath. To assess whether trophic levels of our species were indicative of their diet in Mo'orea, we investigated the relationship between trophic level and nitrogen stable isotope ratio (δ^15^N), which represents diet over longer periods of time (Hesslein et al., 1993). Using δ^15^N values available for a subset of species (*n* = 83) from Mo'orea, we found a strong positive relationship between δ^15^N and trophic level after accounting for body size and phylogenetic relationships (see Appendix S1). These results are consistent with previous observations (Kline & Pauly, 1998) and suggest that food item‐based trophic level is a reasonable indicator of diet, thus supporting its use in our analysis.

FishBase was also used to retrieve species‐level data on body elongation (i.e., standard length divided by maximum body depth). Similar to trophic level, when elongation was unavailable, the mean value of the genus (*n* = 1) was used. We used body elongation to account for body shape as it is the major axis of body shape variation among reef fishes (Claverie & Wainwright, 2014). Moreover, body elongation is strongly related to abdominal cavity depth and the space available to accommodate the intestine and other organs (Burns, 2021).

### 2.2 Data analysis

To investigate the relative contribution of phylogeny, body morphology, and diet in determining intestinal traits, we fitted Bayesian phylogenetic hierarchical linear models. We extracted the phylogeny for the 142 species sampled in Mo'orea from the Fish Tree of Life (Rabosky et al., 2018) using the R package *fishtree* version 0.3.2 (Chang et al., 2019). For species without verified phylogenetic information (*n* = 3), we used the *fishtree_complete_phylogeny()* function to retrieve the pseudo‐posterior distribution of 100 synthetic stochastically resolved phylogenies, with missing species placed using stochastic polytomy resolution.

Using this phylogenetic information, we constructed a phylogenetic relatedness matrix (Hadfield & Nakagawa, 2010) and we tested whether phylogeny, body size, trophic level, body elongation, the presence/absence of the stomach, and a durophagous diet explain intestinal traits using Bayesian phylogenetic hierarchical linear models. To account for both inter and intraspecific scaling, we included a fixed slope on the average measured standard length (SL) per species (i.e., the interspecific variance of SL) and a random slope on the species‐mean‐centered SL (i.e., the individual SL minus the average SL of the species; the intraspecific variance of SL). We also included an interaction term between stomach and durophagy to obtain an estimate for each of the four possible combinations. Thus, the intestinal trait of the *i*th individual of the *j*th species is estimated as follows:

(2) $$\ln{\left(y\right)}_{\mathrm{ij}}=\beta_{0j}+\beta_{1}{\bar{\ln\left(\text{SL}\right)}}_{j}+\beta_{2}{\text{TL}}_{j}+\beta_{3j}\left(\ln{\left(\text{SL}\right)}_{\mathrm{ij}}‐{\bar{\ln\left(\text{SL}\right)}}_{j}\right)+\beta_{4}\ln{\left(\text{EL}\right)}_{j}+\beta_{5}{\text{ST}}_{j}+\beta_{6}{\text{DU}}_{j}+\beta_{7}\text{ST}\times {\text{DU}}_{j},$$

with *β_0j_* and *β_3j_* defined as:

(3) $$\beta_{0j}=\gamma_{00}+u_{0\text{phy}}+u_{0j},$$

(4) $$\beta_{3j}=\gamma_{30}+u_{3j},$$

where *γ*_00_ is the estimated average intercept, *u*_0phy_ and *u*_0_ *_j_* represent deviations from the model intercept attributable to species‐level variation related and unrelated to the phylogeny, respectively, *β_1_* and *β_2_* are the slopes of the species‐mean SL and trophic level (TL), respectively, *γ_30_* is the average slope for the species‐mean‐centered SL, *u_3j_* represents deviations from *γ_30_* attributable to species‐level variation, *β_4_*, *β_5_*, and *β_6_* are the slopes for the body elongation (EL), stomach presence (ST), and durophagous diet (DU), respectively, and *β_7_* is the slope of the interaction between stomach and durophagy. All intestinal traits, fish SL, and elongation were natural‐log‐transformed prior to the analyses. All continuous predictors were centered and scaled to provide a meaningful interpretation of the intercept (i.e., it represents the intestinal trait at the mean body size, trophic level, and elongation for stomachless, non‐durophagous species) and allow comparison between the slopes.

For each intestinal trait, we mapped the predicted mean values onto a phylogenetic tree, including the 139 species with verified phylogenetic positions, using the R package *ggtree* version 2.2.4 (Yu et al., 2017). We further visualized the predicted intestinal traits in two‐dimensional morphospace to characterize the length and diameter of fish intestines and observe the partitioning of intestinal morphology among reef fish families and trophic guilds, which were determined using an unbiased, reproducible trophic categorization scheme (Parravicini et al., 2020). In both the phylogenetic tree and morphospace, parrotfishes (Labridae: tribe Scarini) are depicted separately from other Labridae species since they occupy distinct trophic niches.

To assess the phylogenetic signal (i.e., the tendency of traits in related species to resemble each other more than in species drawn at random from the same tree), we calculated the phylogenetic heritability index, *H^2^*, which is defined as the ratio of the phylogenetic component to the total variance (Lynch, 1991) and is equivalent to Pagel's *λ* (Pagel, 1999). As such, values can vary between zero, for traits that have no phylogenetic component, and one, for traits evolving according to a Brownian motion (random walk) process (Nakagawa & Santos, 2012).

To investigate the intraspecific scaling of intestinal traits, we extracted the random effects on the slopes from our models, which describe their relationship with body size for each species. From the 142 species‐specific slopes for each intestinal trait, we retained those with a 95% credible interval (CI) above zero that belong to species with a minimum of ten sampled individuals whose size range covered at least 25% of the reported maximum body size (retrieved from FishBase). This threshold is necessary to provide reliable estimates of scaling parameters. Isometric scaling (i.e., a proportional relationship with body size during growth) for intestinal length and diameter is defined by a slope of *β* = 1 and for intestinal surface area the slope is *β* = 2. Conversely, slopes that deviate from isometry represent allometric relationships. Thus, slopes below these defined values have negative allometry and slopes above them have positive allometry.

To assess the robustness of the results despite intraspecific variability in morphological traits, we used a sensitivity procedure. All analyses were repeated using two subsets of the complete dataset: (1) 122 species with a minimum of five sampled individuals per species and (2) 69 species with a minimum of eight sampled individuals per species.

### 2.3 Model specifications

**FIGURE 1:**
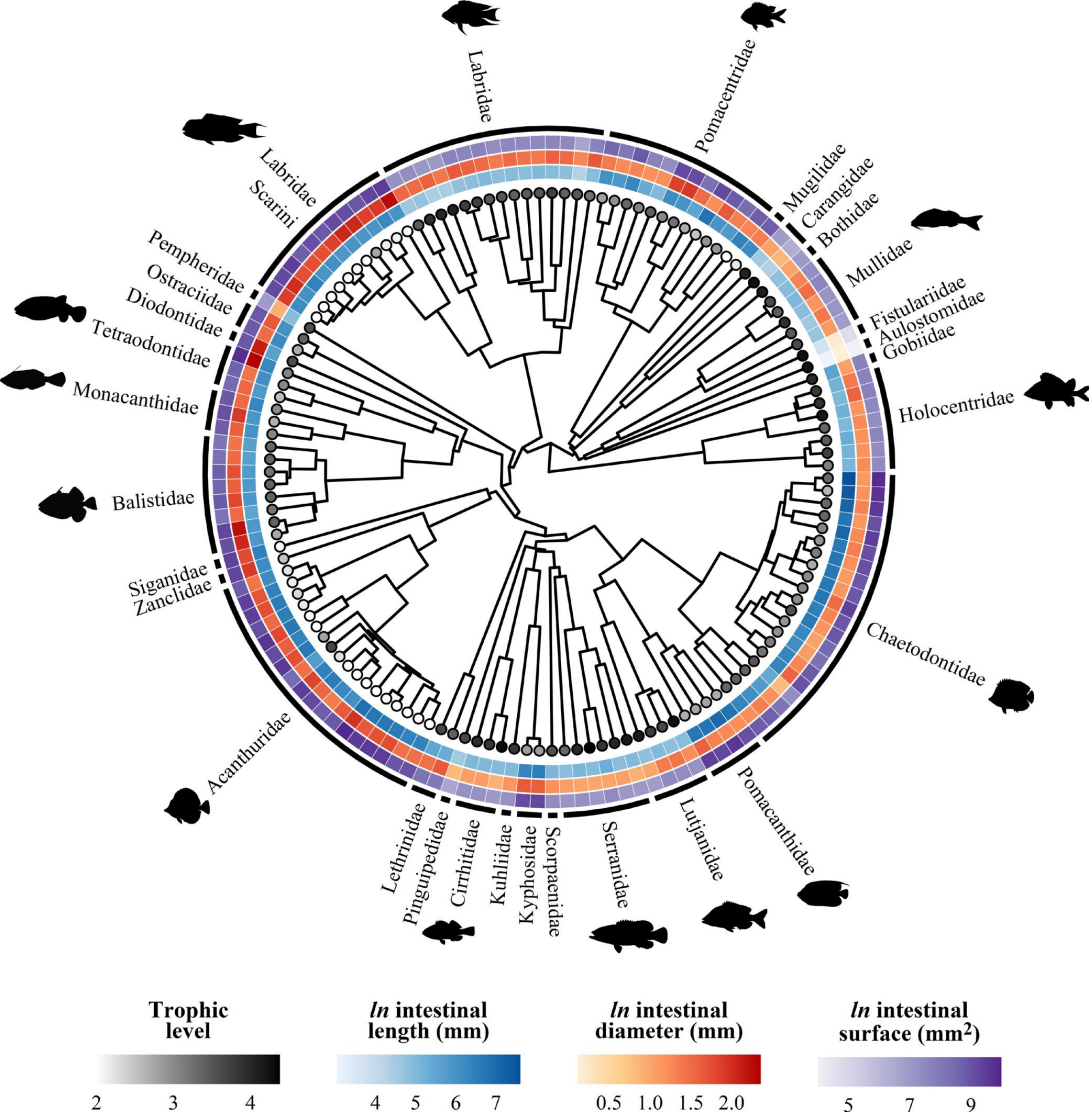
Phylogenetic reconstruction of the 139 reef fish species collected in Mo'orea (generated from the Fish Tree of Life, Chang et al., 2019) with each surrounding ring indicating mean fitted intestinal traits at a standardized fish standard length (SL = 15 cm). Intestinal trait predictions were obtained from Bayesian phylogenetic hierarchical linear models. Colored tip points represent species’ trophic level. Each external arc represents a reef fish family, with silhouettes included for the most speciose families (sourced from Schiettekatte et al., 2019)

We fitted equation 2 using the R package *brms* version 2.14.4 (Bürkner, 2017) to derive posterior distributions and associated 95% CIs for the fitted parameters. We used a Student‐t error distribution and weakly informative, normally distributed priors with means of zero: *N*(0, 10) for the intercept and *N*(0, 5) for fixed effects and species‐level deviations from model intercept and species‐mean‐centered SL mean slope. The posterior distributions of model parameters were estimated using Markov chain Monte Carlo (MCMC) methods by constructing four chains of 8,000 steps with a warm‐up of 2,000 steps. For all models, we inspected the MCMC chains for convergence and model fit (Appendix S2: Figure 1). We used Bayesian *R^2^* to estimate the amount of explained variation from each model (Gelman et al., 2019). All analyses were performed in the software program R (version 4.0.2; R core team, 2020).

## 3 RESULTS

### 3.1 Phylogenetic conservatism

We detected evidence for phylogenetic signal for all intestinal traits. However, phylogeny accounted for a higher variability in intestinal length and surface area (*H^2^* = 0.90 [0.80, 0.94] and *H^2^* = 0.76 [0.50, 0.90], respectively, mean and 95% CI) than intestinal diameter (*H^2^* = 0.34 [0.12, 0.59]). Intestinal morphology varies markedly across the phylogenetic tree, with increases in intestinal length and/or diameter, and, consequently, in surface area, occurring across different lineages (Figure 1). For example, long intestines evolved independently in Acanthuridae, Chaetodontidae, Pomacanthidae, and the tribe Scarini in the Labridae.

### 3.2 Partitioning of intestinal morphology

**FIGURE 2:**
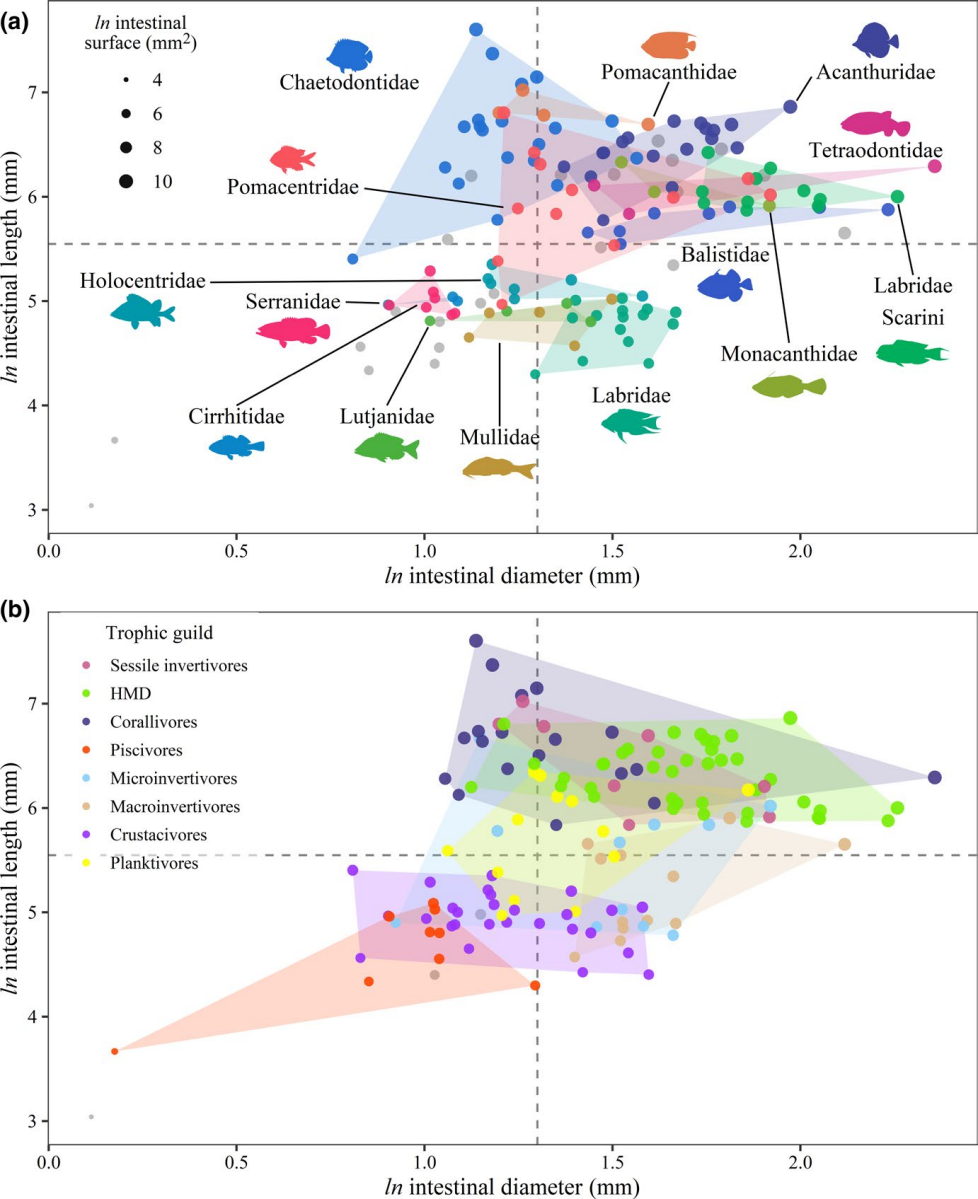
Partitioning of intestinal morphology among (a) reef fish families and (b) trophic guilds (as predicted by Parravicini et al., 2020). Dots (i.e., species) (*n* = 142) are ordered in a morphospace based on intestinal length and diameter and are size‐coded to represent variation in intestinal surface area. Intestinal traits are mean fitted values at a standardized fish standard length (SL = 15 cm), estimated through Bayesian phylogenetic hierarchical linear models. Dashed lines represent the estimated average intestinal length and diameter of non‐durophagous fishes with a stomach (model intercept at SL = 15 cm). Colored polygons show the minimum convex hull plotted per (a) family and (b) trophic guild. Dots are colored according to (a) families represented by at least three species and for which a convex hull could be drawn (for clarity of presentation) and (b) trophic guilds. Gray dots depict (a) species (*n* = 22) belonging to families represented by less than three species and (b) species (*n* = 3) for which Parravicini et al. (2020) did not predict a trophic guild. Fish silhouettes were sourced from Schiettekatte et al., (2019). HMD, herbivores, microvores, and detritivores

The distribution of species based on intestinal morphology (Figure 2) marks a continuum that ranges from short and narrow intestines (piscivores; e.g., *Cephalopholis argus*, Serranidae: 14.26 cm and 0.25 cm, mean estimates of intestinal length and diameter at SL = 15 cm) to long and wide intestines (herbivores; e.g., *Acanthurus guttatus*, Acanthuridae: 95.55 cm and 0.72 cm, mean estimates of intestinal length and diameter at SL = 15 cm). Some species also have short and wide intestines (e.g., invertivorous wrasses, Labridae) or long and narrow intestines (e.g., corallivorous butterflyfishes, Chaetodontidae).

Fish families vary in their distribution across the intestine morphospace and the clearest separation occurs between Acanthuridae, Chaetodontidae, Serranidae, and Labridae (non‐Scarini) which have four distinct intestinal morphologies (i.e., each of these families occupy one of the four quadrants of morphospace; Figure 2a). However, within‐family variation drives overlaps among certain families. Labridae is the most extreme example and presents a clear distinction in intestinal morphology between parrotfishes (Labridae: tribe Scarini) and other wrasses. Conversely, other families with a comparable sample size (e.g., Acanthuridae) have lower within‐family variation in intestinal morphology.

Overlaps are also visible among trophic guilds, despite notable differences in intestinal length (Figure 2b). Herbivores, corallivores, and sessile invertivores have longer intestines than crustacivores and piscivores, while the other trophic guilds have an intermediate intestinal length. Moreover, piscivores generally have a narrower intestine than fishes belonging to other trophic guilds.

### 3.3 Interspecific scaling and relationships with body shape and trophic level

**FIGURE 3:**
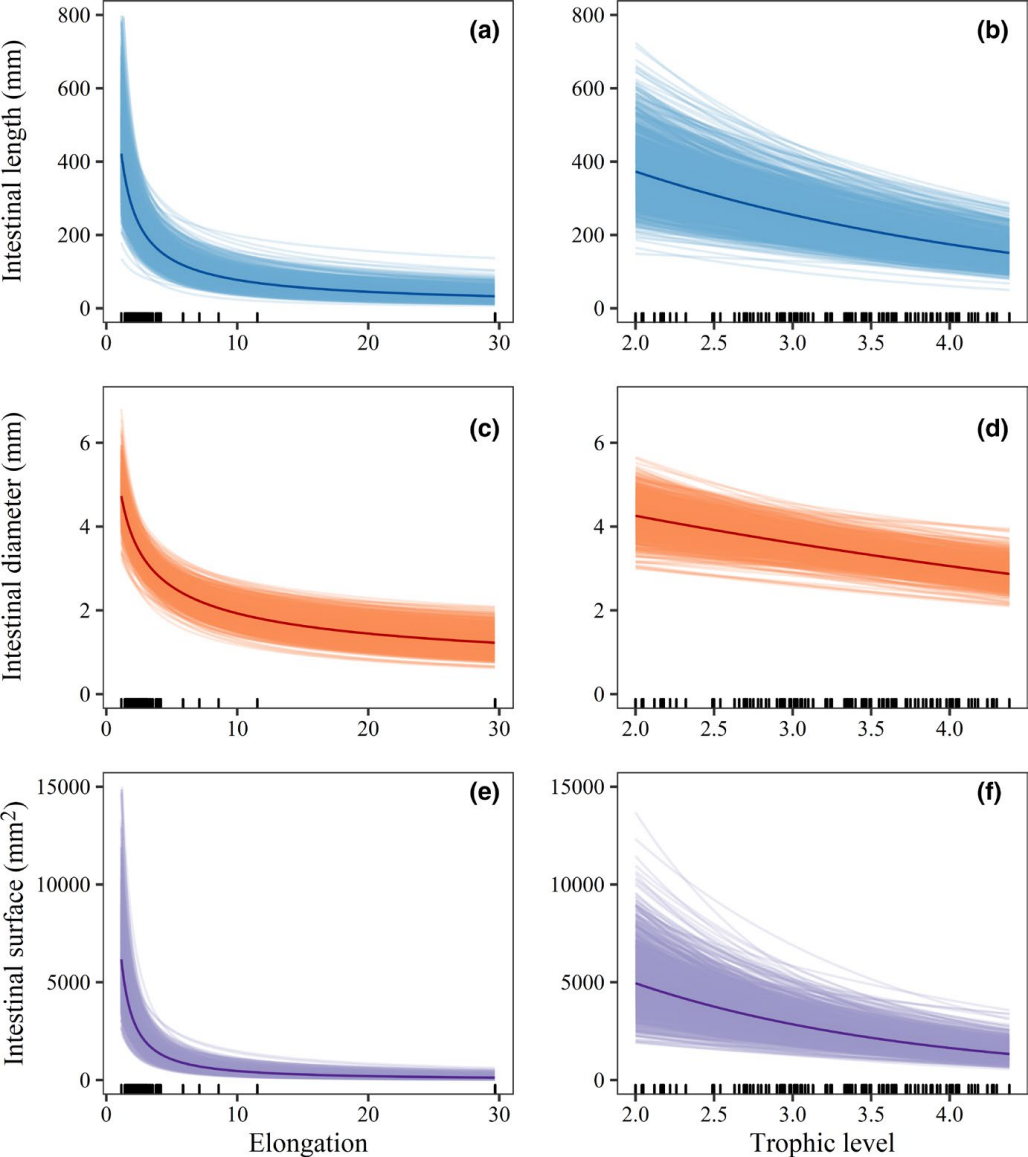
Relationship between three intestinal traits and body elongation (a, c, e) and trophic level (b, d, f) for 142 species of coral reef fishes. Thick, darkened lines represent the mean predicted fits of Bayesian phylogenetic hierarchical linear models after controlling for the remaining fixed and random effects. Categorical variables were set to their most common value (stomach = present, durophagy = non‐durophagous). Thin lines represent 1,000 draws randomly chosen from the posterior fits and show model fit uncertainty. Model predictions are for natural‐log intestinal traits, but are transformed here to show the fitted function on the original scale of the data. Raw data are displayed as marks along the x‐axis

Our model (Eq. 2) explained 92% of the variation in intestinal length and surface area and 85% of the variation in intestinal diameter. Species mean SL consistently had the highest absolute effect size across all intestinal traits (Appendix S2: Table 2) and all traits scaled isometrically across species, with a tendency toward negative allometry for intestinal diameter and surface area (intestinal length: *β* = 0.97 [0.82, 1.13]; intestinal diameter: *β* = 0.93 [0.83, 1.03]; intestinal surface: *β* = 1.87 [1.65, 2.09], mean and 95% CI). After accounting for the other fixed and random effects, all traits decreased with body elongation (intestinal length: *β* = −0.78 [−1.05, −0.52]; intestinal diameter: *β* = −0.42 [−0.56, −0.28]; intestinal surface: *β* = −1.20 [−1.54, −0.85]; Figure 3a,c,e). Additionally, all intestinal traits decreased with trophic level (intestinal length: *β* = −0.38 [−0.53, −0.24]; intestinal diameter: *β* = −0.17 [−0.25, −0.07]; intestinal surface: *β* = −0.55 [−0.81, −0.31]; Figure 3b,d,f), showing a decrease of 59.5% in intestinal length, 32.5% in intestinal diameter and 72.9% in intestinal surface area over the observed trophic levels (from 2.00 to 4.38). The sensitivity analysis confirmed the robustness of the results, even when models were fitted with <50% of the species (Appendix S2: Tables 3–4 and Figure  2).

### 3.4 Influence of stomach presence and durophagy

**FIGURE 4:**
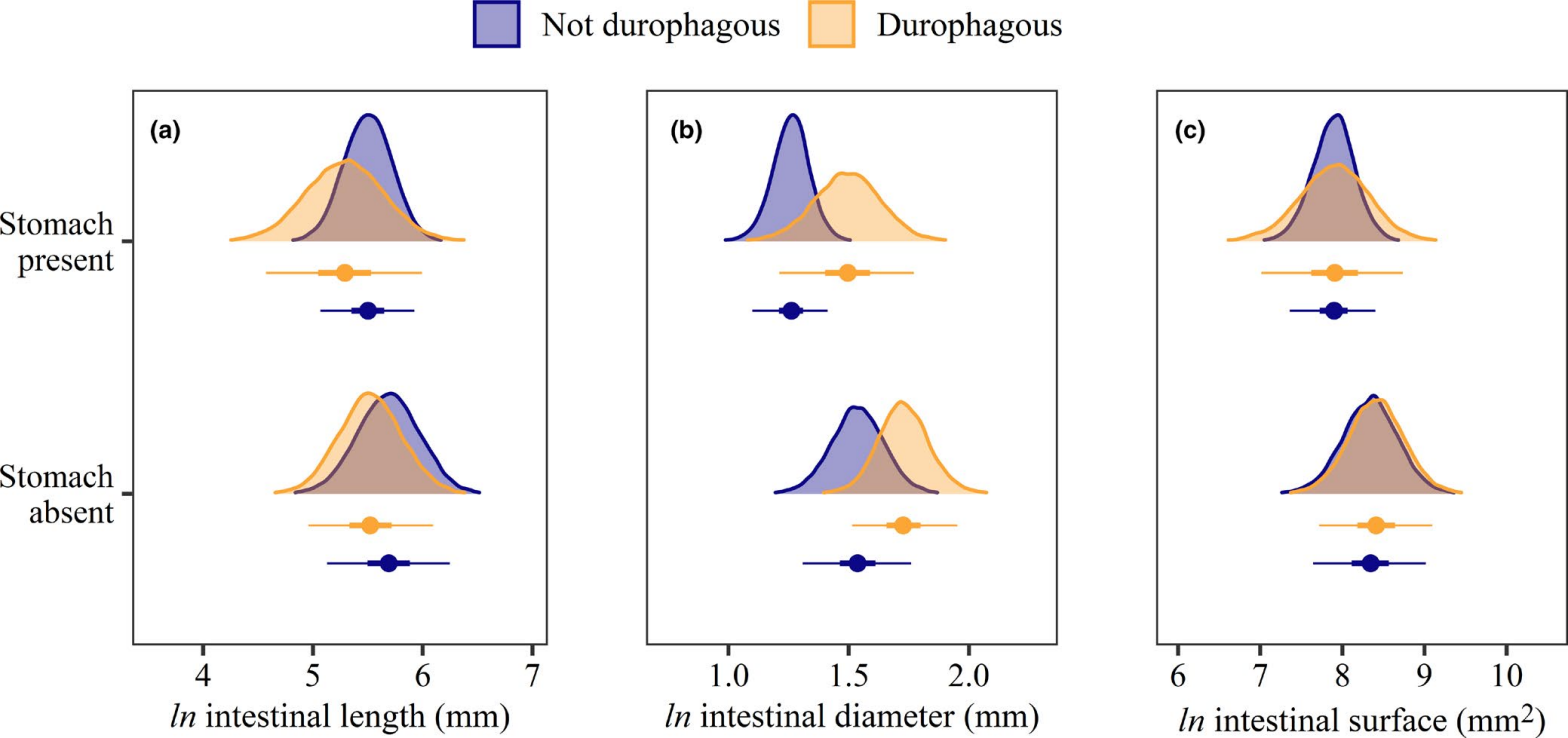
Effects of a stomach and durophagous diet on (a) intestinal length, (b) diameter, and (c) surface area for 142 species of coral reef fishes. Estimates are posterior medians (circles), 50% credible intervals (CIs; thick lines) and 95% CIs (thin lines) from Bayesian phylogenetic hierarchical linear models after controlling for the remaining fixed and random effects. Posterior densities are also displayed (shaded regions)

The presence of a functional stomach and a durophagous diet did not show any interactive effect on intestinal morphology (Figure 4). However, durophagous fishes had a slightly shorter and wider intestine than nondurophagous fishes, irrespective of stomach presence, which resulted in no difference in intestinal surface area. Conversely, fishes with a stomach had a slightly shorter and narrower intestine than stomachless fishes, irrespective of diet. Thus, stomachless fishes had, on average a larger intestinal surface area. The most noticeable difference was observed between stomachless, durophagous species and fishes with a stomach and a non‐durophagous diet, with the former having a larger intestinal diameter. These results remained consistent under our sensitivity analysis (Appendix S2, Figure  3).

### 3.5 Intraspecific scaling

**FIGURE 5:**
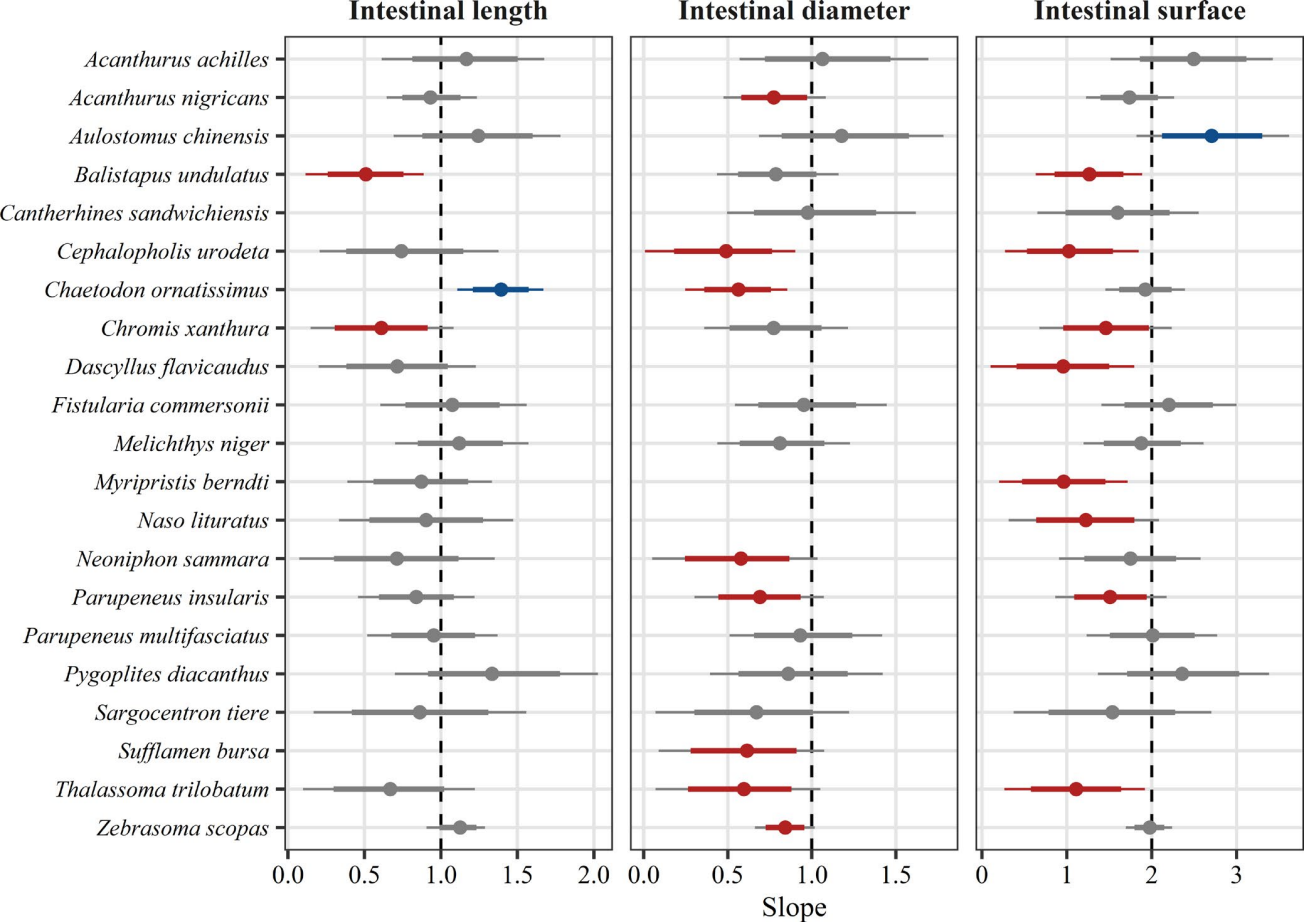
Species‐specific scaling parameters of three natural‐log intestinal traits against natural‐log fish standard length for 21 species of coral reef fishes. Estimates are posterior medians (circles), 80% credible intervals (CIs; thick lines) and 95% CIs (thin lines) from Bayesian phylogenetic hierarchical linear models. Vertical dashed lines represent isometric scaling (*β* = 1 for intestinal length and diameter; *β* = 2 for intestinal surface area). Colored intervals indicate allometric scaling, indicating that more than 90% (if 80% CIs) or 97.5% (if 95% CIs) of the posterior density was either above (blue; positive allometry) or below (red; negative allometry) the isometric scaling parameter, whereas gray intervals indicate that they overlap the parameter. For each trait, species were selected based on a minimum sample size of ten individuals whose size range covered at least 25% of the reported maximum body size (retrieved from FishBase) and a posterior 95% CI above zero to provide reliable estimates of scaling parameters. This selection resulted in missing estimates for one or two traits in five species

From the 142 species‐specific scaling parameters obtained for each intestinal trait (Appendix S2: Table 5), our selection resulted in 19 reliable estimates for intestinal length, 18 for intestinal diameter, and 20 for intestinal surface area (Figure 5). Considering the 80% CIs, three species (16%) showed allometric scaling of intestinal length, including two negative (*Balistapus undulatus*: *β* = 0.51 (0.26, 0.75); *Chromis xanthura*: *β* = 0.61 (0.31, 0.91); median and 80% CI) and one positive allometric relationships (*Chaetodon ornatissimus*: *β* = 1.40 (1.21, 1.57); median and 80% CI). Although no species had positive allometry for intestinal diameter, a negative allometry was found for eight out of 18 species (44%). Lastly, intestinal surface area exhibited allometry in nine species, including one positive scaling (*Aulostomus chinensis*: *β* = 2.72 (2.13, 3.32); median and 80% CI). The remaining 11 species did not deviate from an isometric relationship (*β* = 2).

## 4 DISCUSSION

We investigated the relationship between reef fish intestinal morphology and phylogeny, body morphology, and diet using a large dataset of 142 species and 1,208 individuals. Our results indicate that, although intestinal traits in coral reef fishes are phylogenetically conserved, they are strongly related to body size, body elongation, and trophic level. Among species, intestinal length, diameter, and surface area are negatively correlated with trophic level and body elongation, and they generally scale isometrically with body size. Similarly, within species they predominantly scale isometrically. Furthermore, our analysis shows that intestinal diameter is related to stomach presence and a durophagous diet.

### 4.1 Phylogenetic conservatism

Reef fish intestinal morphology exhibits a high degree of phylogenetic conservatism. The strong phylogenetic signal observed for intestinal length is consistent with previous studies on fishes of the family Cichlidae and Terapontidae (Davis et al., 2013; Wagner et al., 2009), but our analysis revealed less conservatism for intestinal diameter. Further, we confirm that convergent evolution of long and/or wide intestines occurred several times across different lineages (Davis & Betancur‐R, 2017; Davis et al., 2013; Wagner et al., 2009). Chaetodontidae, Pomacanthidae, and herbivorous taxa evolved long intestines with large surface area to exploit trophic niches with nutritionally poor food resources. Conversely, Labridae and Tetraodontiformes, which generally lack a true stomach (Fagundes et al., 2016; Ray & Ringø, 2014; Wilson & Castro, 2010), evolved wide intestines to overcome limitations arising from the lack of food storage and processing inside the stomach. Furthermore, these species have a durophagous diet and well‐developed teeth and/or pharyngeal jaws that grind food into smaller fragments, partly replacing the function of the stomach (Gromova & Maktotin, 2019; Wainwright et al., 2012). The size of these particles remains, however, too large to be funneled through a thin intestine and may require a thicker intestinal wall to protect from mechanical damage (Fagundes et al., 2016). Although we cannot discern whether the large external intestinal diameter in Labridae and Tetraodontiformes is the result of a wider intestinal lumen, thicker intestinal wall, or a combination of the two, it appears that wide intestines have evolved multiple times, along with specializations of the feeding apparatus (Wainwright et al., 2012), to exploit trophic resources otherwise unattainable.

Additionally, phylogenetic conservatism can be clearly observed within the Labridae. Within this family, parrotfishes (Labridae: tribe Scarini) have a larger intestine than other species. Evolutionary history has mainly led to an increase in the intestinal length and surface area in parrotfishes; however, intestinal diameter is conserved at the family level (see Appendix S2: Figure 4). The large intestine, together with cranial specializations (Gobalet, 2018), could have played a substantial role in the initial divergence of the Scarini clade (Streelman et al., 2002), allowing them to adapt to an herbivorous diet and diversify rapidly (Siqueira et al., 2020), whereas other wrasses remained carnivores (Cowman et al., 2009; Floeter et al., 2018).

### 4.2 Interspecific scaling and relationships with body shape and trophic level

Among coral reef fishes, intestinal traits scale isometrically with body size after accounting for variation in body shape. Correction for body shape, in addition to allometry, is important because larger fishes have long body plans (Friedman et al., 2019), which in turn have relatively small abdominal cavities (Burns, 2021) that may not accommodate large intestines. For instance, the two most distinctively elongated species in our dataset, *Aulostomus chinensis* and *Fistularia commersonii*, both of which are strict piscivores, had the lowest values across all intestinal traits.

Regardless of taxonomic identity, body size, and elongation, trophic level strongly influences intestinal morphology. The negative relationship between intestinal length and trophic level is consistent with previous work on marine and freshwater fishes (Elliott & Bellwood, 2003; Kramer & Bryant, 1995b; Wagner et al., 2009). Furthermore, we found that the same negative relationship holds true for other intestinal traits, providing the first quantitative evidence that intestinal diameter, as well as length, varies as a function of trophic level. However, while carnivores and herbivores have the widest intestine and corallivores the narrowest across three reef fish families (Elliott & Bellwood, 2003), a clear relationship between intestinal diameter and diet has not yet been established. We observed a significant decrease in diameter with increasing trophic level. Fishes with the highest trophic level (4.38) had a 32.5% narrower intestine than herbivorous fishes. Beyond the larger number of families sampled here, using a continuous variable to delineate reef fish diet (i.e., trophic level) helped uncover this relationship as opposed to the use of categorical trophic groups (Elliott & Bellwood, 2003). These results suggest that intestinal diameter is useful to further delineate fish diet partitioning, and intestinal surface area, which incorporates variability in both length and diameter, may be a better descriptor of interspecific differences in intestinal morphology than intestinal length alone.

On average, the predicted intestinal surface area of herbivorous fishes in Mo'orea was four times that of fishes that occupy the highest trophic level. While this difference is determined by the increase in both intestinal length and diameter, the different rate of variation in these traits leads to intestinal elongation with decreasing trophic level. This increases the intestinal surface available for the absorption of nutrients, but it also increases food retention time, which is known to favor the digestion of food with low nutritional quality (Lassuy, 1984; Sibly, 1981).

### 4.3 Intraspecific scaling

In the present study, we provide estimates of scaling parameters for at least one intestinal trait of 21 reef fish species. Our results show that two thirds of these species exhibit allometric scaling in one or more traits, with several taxa decreasing the relative size of their intestinal diameter or surface area with increasing body size. For the remaining species, our data do not show any significant deviation from isometry. Widespread allometric elongation of the intestine has been observed in both marine and freshwater fishes (Karachle & Stergiou, 2010; Kramer & Bryant, 1995a; Ribble & Smith, 1983). In contrast, we found positive allometry in intestinal length only for *C*. *ornatissimus*, while most species showed isometry. Our results highlight a tendency toward negative allometry in intestinal diameter and surface area. Thus, while the relative length of the intestine may remain constant or increase throughout an individual's lifetime, it generally becomes narrower and decreases in surface area. These results are consistent with the decrease in relative intestinal surface area observed in some herbivorous fishes (Al‐Hussaini, 1949; Gohar & Latif, 1959; Horn, 1989; Montgomery, 1977) and the negative allometry in intestinal mass and metabolic capacity reported for two species of Cyprinidae (Goolish & Adelman, 1988) and are potentially related to decreases in growth with increased size.

### 4.4 Intestinal morphology and function

Our results highlight the tight link between intestinal morphology and the digestive and assimilating functions in reef fishes. Intestinal traits are clear indicators of fish trophic roles and thus suitable for trait‐based ecological research (Villéger et al., 2017). While intestinal length is commonly used in fish functional studies (Mouchet et al., 2013; Villéger et al., 2010; Zhao et al., 2019), we show that intestinal diameter provides an important addition to better segregate fish dietary habits and should therefore be considered. The intestine also plays an important role in other fish functions, such as the absorption of nutrients (Crossman et al., 2005) and carbonate excretion (Wilson et al., 2002), which are key contributors to nutrient cycling and inorganic carbon cycling. Therefore, the intestinal traits presented herein could be used to explore relationship with these functions in future studies.

In the present study, we mainly focused on interspecific variability in intestinal morphology. However, in Mo'orea, the fishes were collected around the entire island, including a great span of habitats (lagoon and slope; coral‐dominated and algae‐dominated reefs), and across multiple seasons. While these variables were not explicitly accounted for in our analysis due to limited replication, our models explained 85% to 92% of the variation in the data, demonstrating that intraspecific variability, independent of body size, was minor compared to interspecific variability in our dataset. Nevertheless, spatial and temporal variation in food availability and/or nutritional quality may lead to intraspecific variability (Olsson et al., 2007; Wagner et al., 2009). Therefore, these factors should be considered in future studies to fully understand the relationship between intestinal morphology and diet and assess the extent of intraspecific variability.

## 5 CONCLUSION

Our findings show that intestinal traits are highly conserved across reef fish phylogeny. We also demonstrate that via adaptive convergent evolution, intestinal flexibility permitted the occupation of trophic niches characterized by the uptake of food resources with low nutritional quality across diverse phylogenetic groups. Further, trophic level is strongly related to intestinal diameter, as well as length, in coral reef fishes. Species that occupy low trophic levels surmount the low nutritional value of food items by increasing intestinal absorptive surface and maximizing nutrient intake. This is achieved with a differential increase in the length and diameter of the intestine, which results in an elongate alimentary tract that prolongs food retention. Thus, for trait‐based ecological studies, intestinal length and diameter should be used together. Alternatively, if using a single trait, intestinal surface area may be a better descriptor of inter and intraspecific differences in diet than intestinal length.

## CONFLICT OF INTEREST

The authors declare no conflict of interest.

## AUTHOR CONTRIBUTION

**Mattia Ghilardi:** Conceptualization (equal); Data curation (lead); Formal analysis (lead); Investigation (equal); Methodology (equal); Software (lead); Visualization (lead); Writing‐original draft (lead); Writing‐review & editing (lead). **Nina M. D. Schiettekatte:** Conceptualization (equal); Data curation (supporting); Formal analysis (supporting); Investigation (equal); Methodology (equal); Writing‐original draft (supporting); Writing‐review & editing (supporting). **Jordan M. Casey:** Investigation (equal); Writing‐review & editing (supporting). **Simon J. Brandl:** Investigation (equal); Writing‐review & editing (supporting). **Samuel Degregori:** Investigation (equal); Writing‐review & editing (supporting). **Alexandre Mercière:** Investigation (equal); Writing‐review & editing (supporting). **Fabien Morat:** Investigation (equal); Writing‐review & editing (supporting). **Yves Letourneur:** Investigation (equal); Writing‐review & editing (supporting). **Sonia Bejarano:** Supervision (supporting); Writing‐review & editing (supporting). **Valeriano Parravicini:** Conceptualization (equal); Funding acquisition (lead); Investigation (equal); Methodology (equal); Project administration (lead); Resources (lead); Supervision (lead); Writing‐original draft (supporting); Writing‐review & editing (supporting).

## Supporting information

Appendix S1

Appendix S2

## Data Availability

All data and code necessary to reproduce the results and figures are publicly available on Zenodo: https://doi.org/10.5281/zenodo.5172790.
