# Supplementary material for: Phylogeny, body morphology, and trophic level shape intestinal traits in coral reef fishes
Source: Ecol Evol. 2021 Aug 29;11(19):13218–31. doi: 10.1002/ece3.8045 (PMC8495780; doi:10.1002/ece3.8045)
Supplement: Supplementary file 1 — Appendix S1 [file ECE3-11-13218-s001.pdf]

Appendix S1: Supplementary methods

Relationship between trophic level and $\delta^{15}N$

To test whether food-item based trophic levels in FishBase are representative of fish diet in Mo'orea we built a dataset of nitrogen stable isotope ratios ($\delta^{15}N$) for 83 of the 142 species present in our dataset of intestinal traits. A total of 700 individuals (n = 3 per species minimum) collected from reefs around Mo'orea, in French Polynesia, including 319 in 2016 and 381 in 2018, were examined. All individuals were collected by spearfishing between 10:00 and 15:00 and transferred to the laboratory in a cooler. In the laboratory, each individual was measured and weighed, and a small piece of dorsal muscle was sampled and immediately frozen at −20°C.

Nitrogen stable isotope data ($\delta^{15}N$) were determined in all samples that were freeze-dried and ground to fine powder. Measurements were done by continuous-flow isotope-ratio mass spectrometry with a NC2500 elemental analyzer (CE Instruments, Wigan, UK), coupled with a Delta V isotope ratio mass spectrometer (Thermo Scientific, Bremen, Germany) at the Cornell University Stable Isotope Laboratory (COIL). Isotope compositions were expressed in the δ notation as parts per mil (‰) as deviations from atmospheric N_2 following the formula:

$$\delta X = [(R_{sample} / R_{standard}) - 1] \, x \, 1000, \quad (1)$$

where X is ^{15}N, R is the corresponding $^{15}N/^{14}N$ ratio. Calibration was done using two in-house standards (CBT and KCRN). The analytical precision of the measurements was 0.24‰ based on analyses of an in-house animal standard (Deer).

To assess the relationship between trophic level and $\delta^{15}N$ we fitted a Bayesian phylogenetic hierarchical linear model. We extracted the phylogeny for the 83 species in the dataset from the Fish Tree of Life (Rabosky et al., 2018) using the R package *fishtree* version 0.3.2 (Chang et al., 2019).

Using this phylogenetic information, we constructed a phylogenetic relatedness matrix (Hadfield & Nakagawa, 2010) and we tested whether body size, trophic level and phylogeny explain the variation in δ^{15}N values using a Bayesian phylogenetic hierarchical linear model. The δ^{15}N value of the ith individual of the jth species is estimated as follows:

$$\delta^{15}N_{ij} = \beta_{0j} + \beta_{1j} ln\,(SL)_{ij} + \beta_2 TL_j, \qquad (2)$$

with β_{0j} and β_{1j} defined as:

$$\beta_{0j} = \gamma_{00} + u_{0phy} + u_{0j}, \qquad (3)$$

$$\beta_{1j} = \gamma_{10} + u_{1j}, \qquad (4)$$

where γ_{00} is the estimated average intercept, u_{0phy} and u_{0j} represent deviations from the model intercept attributable to species-level variation related and unrelated to the phylogeny, respectively, γ_{10} is the average slope for the natural-log transformed standard length (SL), u_{1j} represents deviations from γ_{10} attributable to species-level variation, and β_2 is the slope for trophic level (TL). All predictors were centred and scaled. We fitted the model with the R package *brms* version 2.14.4 (Bürkner, 2017) using a student-t error distribution. We used normally distributed priors with a mean of zero for intercept, fixed and random effects, and ran the model for four chains, each with 4,000 steps and a warm-up of 1,000 steps.

Our model explained 82.2% of the variation in δ^{15}N values across species. Nitrogen isotope ratios were influenced by phylogeny (H^2 = 0.74 [0.40, 0.94], mean and 95% credible interval). After controlling for the phylogenetic relationships, δ^{15}N was positively related to body size (β = 0.79 [0.45, 1.14]) and trophic level (β = 0.97 [0.42, 1.51]), and the latter explained the highest proportion of variance in δ^{15}N (highest standardised effect; Table 1). Nitrogen isotope ratios increased linearly with

trophic level (one unit increase in $\delta^{15}N$ for every one unit increase in trophic level on average), implying a 27.6% increase in $\delta^{15}N$ over the observed range of trophic level (from 2.00 to 4.38; Fig. 1). Although on average $\delta^{15}N$ was positively related to body size, this relationship varied greatly among species (Table 1).

Table 1 Posterior mean and 95% credible interval for each parameter of a Bayesian phylogenetic hierarchical linear model of $\delta^{15}N$ for 83 species of coral reef fishes. α = model intercept; β_{SL} = scaled log standard length slope; β_{TL} = scaled trophic level slope; σ_{phy} = random intercept standard deviation, phylogenetic relationship; σ_{sp} = random intercept standard deviation, species; $\sigma_{\beta sp}$ = random slope standard deviation, species; σ_{ϵ} = residual standard deviation; ν = degrees of freedom of student-t distribution.

**parameter**	**mean**	**2.5%**	**97.5%**
α	9.51	8.44	10.53
β_{SL}	0.40	0.22	0.57
β_{TL}	0.66	0.29	1.03
σ_{phy}	1.33	0.79	1.90
σ_{sp}	0.53	0.06	0.91
$\sigma_{\beta sp}$	0.47	0.31	0.66
σ_{ϵ}	0.47	0.41	0.53
ν	2.81	2.11	3.76

Fig. 1 Relationship between $\delta^{15}N$ and trophic level for 83 species of coral reef fishes. Thick, darkened line represents the mean predicted fit of a Bayesian phylogenetic hierarchical linear model after controlling for body size and phylogeny. Thin lines represent 1,000 draws randomly chosen from the posterior fits and show model fit uncertainty. Dots represent the raw values.

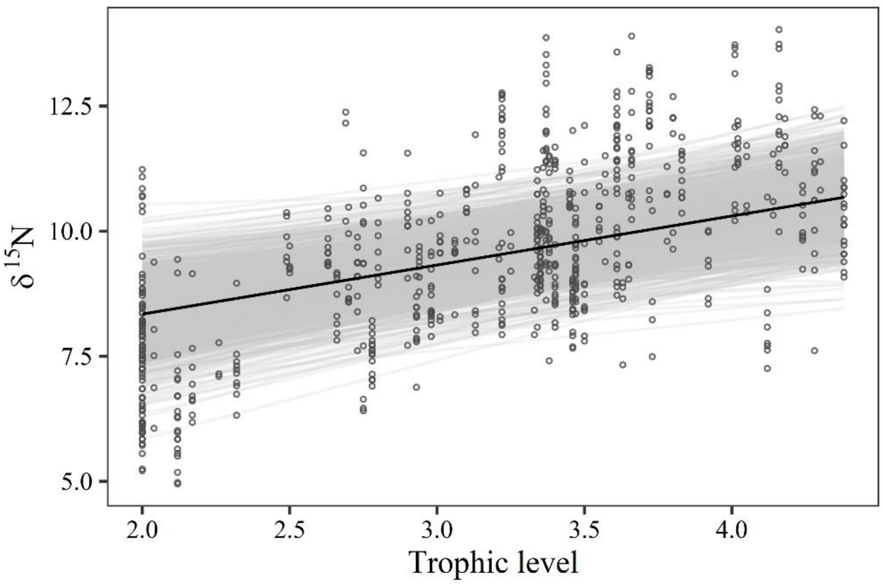

References

Bürkner, P.-C. (2017). Brms : an R package for bayesian multilevel models using Stan. *Journal of Statistical Software*, 80(1). https://doi.org/10.18637/jss.v080.i01

Chang, J., Rabosky, D. L., Smith, S. A., & Alfaro, M. E. (2019). An R package and online resource for macroevolutionary studies using the ray-finned fish tree of life. *Methods in Ecology and Evolution*, 10, 1118–1124. https://doi.org/10.1111/2041-210X.13182

Hadfield, J. D., & Nakagawa, S. (2010). General quantitative genetic methods for comparative biology: phylogenies, taxonomies and multi-trait models for continuous and categorical characters. *Journal of Evolutionary Biology*, 23(3), 494–508. https://doi.org/10.1111/j.1420-9101.2009.01915.x

Rabosky, D. L., Chang, J., Title, P. O., Cowman, P. F., Sallan, L., Friedman, M., Kaschner, K., Garilao, C., Near, T. J., Coll, M., & Alfaro, M. E. (2018). An inverse latitudinal gradient in speciation rate for marine fishes. *Nature*, 559(7714), 392–395. https://doi.org/10.1038/s41586-018-0273-1
