# Supplementary material for: Phylogeny, body morphology, and trophic level shape intestinal traits in coral reef fishes
Source: Ecol Evol. 2021 Aug 29;11(19):13218–31. doi: 10.1002/ece3.8045 (PMC8495780; doi:10.1002/ece3.8045)
Supplement: Supplementary file 2 — Appendix S2 [file ECE3-11-13218-s002.pdf]

Appendix S2: supplementary tables and figures

Table 1 Number of individuals per species collected from three different habitats.

Family	Species	Slope	Lagoon	Pass	Total
Acanthuridae	*Acanthurus achilles*	12	0	0	12
Acanthuridae	*Acanthurus guttatus*	8	1	0	9
Acanthuridae	*Acanthurus lineatus*	7	0	2	9
Acanthuridae	*Acanthurus nigricans*	12	1	0	13
Acanthuridae	*Acanthurus nigrofuscus*	2	3	1	6
Acanthuridae	*Acanthurus nigros*	3	0	1	4
Acanthuridae	*Acanthurus olivaceus*	5	0	1	6
Acanthuridae	*Acanthurus pyroferus*	2	2	1	5
Acanthuridae	*Acanthurus thompsoni*	3	0	1	4
Acanthuridae	*Acanthurus triostegus*	5	8	2	15
Acanthuridae	*Ctenochaetus flavicauda*	4	0	2	6
Acanthuridae	*Ctenochaetus striatus*	2	3	0	5
Acanthuridae	*Naso brevirostris*	6	0	0	6
Acanthuridae	*Naso lituratus*	17	0	0	17
Acanthuridae	*Naso unicornis*	1	0	4	5
Acanthuridae	*Naso vlamingii*	3	0	2	5
Acanthuridae	*Zebrasoma scopas*	26	16	0	42
Acanthuridae	*Zebrasoma velifer*	4	0	5	9
Aulostomidae	*Aulostomus chinensis*	3	7	1	11
Balistidae	*Balistapus undulatus*	8	4	0	12
Balistidae	*Melichthys niger*	14	0	0	14
Balistidae	*Melichthys vidua*	10	2	0	12
Balistidae	*Odonus niger*	11	3	0	14
Balistidae	*Rhinecanthus aculeatus*	0	7	2	9
Balistidae	*Rhinecanthus lunula*	2	0	1	3
Balistidae	*Rhinecanthus rectangulus*	6	0	3	9
Balistidae	*Sufflamen bursa*	6	6	0	12
Blenniidae	*Exallias brevis*	3	0	2	5
Bothidae	*Bothus mancus*	0	1	2	3
Carangidae	*Caranx melampygus*	4	0	1	5
Carangidae	*Scomberoides lysan*	2	0	1	3
Chaetodontidae	*Chaetodon auriga*	2	2	5	9
Chaetodontidae	*Chaetodon bennetti*	0	0	3	3
Chaetodontidae	*Chaetodon citrinellus*	3	2	2	7
Chaetodontidae	*Chaetodon ephippium*	3	4	1	8
Chaetodontidae	*Chaetodon lunula*	1	4	4	9
Chaetodontidae	*Chaetodon lunulatus*	0	8	0	8

Family	Species	Slope	Lagoon	Pass	Total
Chaetodontidae	*Chaetodon mertensii*	0	1	4	5
Chaetodontidae	*Chaetodon ornatissimus*	29	0	0	29
Chaetodontidae	*Chaetodon pelewensis*	6	0	0	6
Chaetodontidae	*Chaetodon quadrimaculatus*	6	0	2	8
Chaetodontidae	*Chaetodon reticulatus*	32	0	0	32
Chaetodontidae	*Chaetodon trichrous*	2	1	3	6
Chaetodontidae	*Chaetodon trifascialis*	2	1	3	6
Chaetodontidae	*Chaetodon ulietensis*	2	1	5	8
Chaetodontidae	*Chaetodon unimaculatus*	5	3	1	9
Chaetodontidae	*Chaetodon vagabundus*	4	9	0	13
Chaetodontidae	*Forcipiger flavissimus*	2	6	0	8
Chaetodontidae	*Forcipiger longirostris*	7	0	0	7
Chaetodontidae	*Hemitaurichthys polylepis*	5	0	2	7
Chaetodontidae	*Heniochus chrysostomus*	1	7	0	8
Cirrhitidae	*Cirrhitus pinnulatus*	6	0	4	10
Cirrhitidae	*Paracirrhites forsteri*	6	1	2	9
Cirrhitidae	*Paracirrhites hemistictus*	17	0	1	18
Diodontidae	*Diodon hystrix*	1	2	2	5
Fistulariidae	*Fistularia commersonii*	1	4	5	10
Gobiidae	*Valenciennea strigata*	0	5	1	6
Hemiramphidae	*Hemiramphus depauperatus*	1	2	0	3
Holocentridae	*Myripristis berndti*	1	15	0	16
Holocentridae	*Myripristis kuntee*	0	6	1	7
Holocentridae	*Myripristis violacea*	0	2	7	9
Holocentridae	*Neoniphon sammara*	0	12	0	12
Holocentridae	*Sargocentron caudimaculatum*	0	7	0	7
Holocentridae	*Sargocentron spiniferum*	5	4	1	10
Holocentridae	*Sargocentron tiere*	5	5	1	11
Kuhliidae	*Kuhlia mugil*	2	0	1	3
Kyphosidae	*Kyphosus cinerascens*	0	0	3	3
Kyphosidae	*Kyphosus vaigiensis*	2	0	2	4
Labridae	*Calotomus carolinus*	3	2	4	9
Labridae	*Cheilinus chlorourus*	0	9	0	9
Labridae	*Cheilinus trilobatus*	1	6	0	7
Labridae	*Cheilio inermis*	0	0	4	4
Labridae	*Chlorurus microrhinos*	4	0	0	4
Labridae	*Chlorurus spilurus*	5	1	0	6
Labridae	*Coris aygula*	3	4	2	9
Labridae	*Coris gaimard*	5	0	2	7
Labridae	*Epibulus insidiator*	12	4	0	16

Family	Species	Slope	Lagoon	Pass	Total
Labridae	*Gomphosus varius*	4	3	0	7
Labridae	*Halichoeres hortulanus*	0	8	1	9
Labridae	*Halichoeres trimaculatus*	0	5	0	5
Labridae	*Hemigymnus fasciatus*	8	0	1	9
Labridae	*Novaculichthys taeniourus*	2	1	3	6
Labridae	*Oxycheilinus unifasciatus*	3	0	1	4
Labridae	*Scarus altipinnis*	6	0	0	6
Labridae	*Scarus forsteni*	8	1	0	9
Labridae	*Scarus frenatus*	2	2	0	4
Labridae	*Scarus globiceps*	1	2	4	7
Labridae	*Scarus oviceps*	6	1	0	7
Labridae	*Scarus psittacus*	0	6	3	9
Labridae	*Scarus rubroviolaceus*	2	0	3	5
Labridae	*Scarus schlegeli*	3	0	4	7
Labridae	*Thalassoma hardwicke*	0	7	0	7
Labridae	*Thalassoma purpureum*	4	1	0	5
Labridae	*Thalassoma trilobatum*	6	1	3	10
Lethrinidae	*Gnathodentex aureolineatus*	1	4	2	7
Lethrinidae	*Monotaxis grandoculis*	4	2	1	7
Lutjanidae	*Aphareus furca*	7	0	2	9
Lutjanidae	*Lutjanus fulvus*	3	6	2	11
Lutjanidae	*Lutjanus kasmira*	6	0	3	9
Lutjanidae	*Lutjanus monostigma*	2	0	3	5
Monacanthidae	*Aluterus scriptus*	0	0	4	4
Monacanthidae	*Amanses scopas*	8	0	1	9
Monacanthidae	*Cantherhines sandwichiensis*	9	0	1	10
Mugilidae	*Ellochelon vaigiensis*	0	2	2	4
Mullidae	*Mulloidichthys flavolineatus*	0	8	1	9
Mullidae	*Mulloidichthys vanicolensis*	1	1	3	5
Mullidae	*Parupeneus barberinus*	0	3	1	4
Mullidae	*Parupeneus insularis*	7	2	2	11
Mullidae	*Parupeneus multifasciatus*	3	8	0	11
Ostraciidae	*Ostracion cubicus*	0	1	2	3
Ostraciidae	*Ostracion meleagris*	0	5	2	7
Pempheridae	*Pempheris oualensis*	6	0	1	7
Pinguipedidae	*Parapercis millepunctata*	0	7	0	7
Pomacanthidae	*Centropyge flavissima*	4	2	2	8
Pomacanthidae	*Centropyge loriculus*	3	0	0	3
Pomacanthidae	*Pomacanthus imperator*	7	0	0	7
Pomacanthidae	*Pygoplites diacanthus*	3	3	4	10

**Family**	**Species**	**Slope**	**Lagoon**	**Pass**	**Total**
Pomacentridae	*Abudefduf septemfasciatus*	1	8	2	11
Pomacentridae	*Abudefduf sexfasciatus*	0	8	1	9
Pomacentridae	*Abudefduf sordidus*	2	1	3	6
Pomacentridae	*Chromis atripectoralis*	0	3	2	5
Pomacentridae	*Chromis xanthura*	22	0	2	24
Pomacentridae	*Dascyllus aruanus*	0	3	4	7
Pomacentridae	*Dascyllus flavicaudus*	9	5	0	14
Pomacentridae	*Dascyllus trimaculatus*	1	5	2	8
Pomacentridae	*Plectroglyphidodon johnstonianus*	6	0	0	6
Pomacentridae	*Plectroglyphidodon lacrymatus*	6	0	0	6
Pomacentridae	*Stegastes fasciolatus*	6	0	3	9
Pomacentridae	*Stegastes nigricans*	0	7	3	10
Scorpaenidae	*Pterois radiata*	3	5	1	9
Serranidae	*Cephalopholis argus*	15	1	1	17
Serranidae	*Cephalopholis urodeta*	13	0	1	14
Serranidae	*Epinephelus fasciatus*	5	0	1	6
Serranidae	*Epinephelus hexagonatus*	5	0	2	7
Serranidae	*Epinephelus merra*	0	8	1	9
Serranidae	*Pseudanthias pascalus*	5	0	2	7
Serranidae	*Variola louti*	6	0	1	7
Siganidae	*Siganus spinus*	2	3	0	5
Tetraodontidae	*Arothron meleagris*	4	5	0	9
Tetraodontidae	*Canthigaster bennetti*	0	3	0	3
Tetraodontidae	*Canthigaster solandri*	0	5	1	6
Zanclidae	*Zanclus cornutus*	8	3	0	11

Fig. 1 Posterior predictive checks based on 100 posterior samples for Bayesian phylogenetic hierarchical linear models of (a) intestinal length, (b) diameter, and (c) surface area of coral reef fishes.

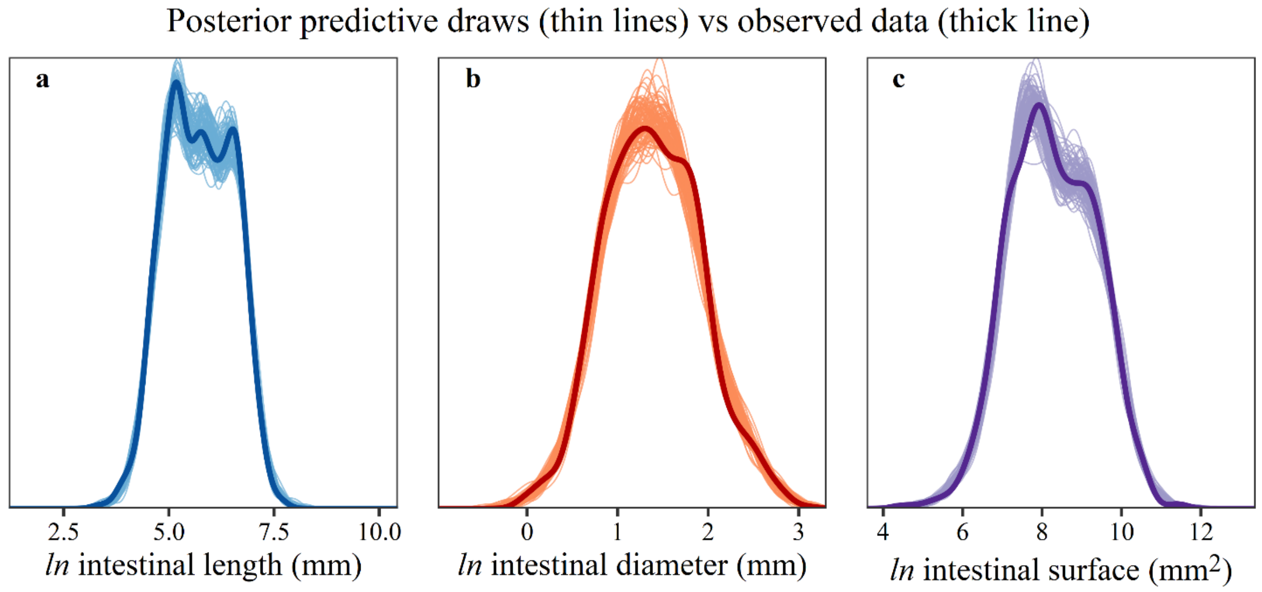

Table 2 Posterior mean and 95% credible interval for each parameter of Bayesian phylogenetic hierarchical linear models of intestinal length, diameter, and surface area for 142 species of coral reef fishes with a minimum sample size of three individuals per species. α = model intercept; $\beta_{SL\text{-}inter}$ = scaled species mean standard length slope; β_{TL} = scaled trophic level slope; $\beta_{SL\text{-}intra}$ = scaled species-mean-centred standard length slope; β_{EL} = scaled elongation slope; β_{ST} = stomach slope; β_{DU} = durophagy slope; $\beta_{ST:DU}$ = stomach X durophagy slope; σ_{phy} = random intercept standard deviation, phylogenetic relationship; σ_{sp} = random intercept standard deviation, species; $\sigma_{\beta sp}$ = random slope standard deviation, species; σ_{ϵ} = residual standard deviation; v = degrees of freedom of student-t distribution.

	**Length**			**Diameter**			**Surface**		
**parameter**	**mean**	**2.5%**	**97.5%**	**mean**	**2.5%**	**97.5%**	**mean**	**2.5%**	**97.5%**
α	5.69	5.13	6.25	1.54	1.31	1.76	8.34	7.64	9.01
$\beta_{SL\text{-}inter}$	0.42	0.35	0.49	0.40	0.36	0.45	0.81	0.71	0.90
β_{TL}	-0.26	-0.36	-0.16	-0.11	-0.17	-0.05	-0.38	-0.55	-0.21
$\beta_{SL\text{-}intra}$	0.13	0.11	0.15	0.11	0.10	0.13	0.24	0.21	0.27
β_{EL}	-0.32	-0.43	-0.21	-0.17	-0.23	-0.11	-0.49	-0.63	-0.35
β_{ST}	-0.19	-0.68	0.30	-0.28	-0.49	-0.07	-0.45	-1.07	0.17
β_{DU}	-0.17	-0.57	0.23	0.19	-0.01	0.40	0.07	-0.43	0.59
$\beta_{ST:DU}$	-0.05	-0.81	0.70	0.04	-0.29	0.37	-0.06	-0.99	0.84
σ_{phy}	0.54	0.44	0.64	0.18	0.10	0.27	0.64	0.42	0.83
σ_{sp}	0.07	0.00	0.16	0.15	0.11	0.19	0.19	0.02	0.33
$\sigma_{\beta sp}$	0.06	0.04	0.08	0.04	0.02	0.07	0.10	0.07	0.13

parameter	Length mean	Length 2.5%	Length 97.5%	Diameter mean	Diameter 2.5%	Diameter 97.5%	Surface mean	Surface 2.5%	Surface 97.5%
σ_{ϵ}	0.16	0.14	0.17	0.19	0.18	0.21	0.28	0.26	0.30
ν	4.52	3.34	6.25	15.96	7.27	37.44	12.51	6.63	26.09

Table 3 Posterior mean and 95% credible interval for each parameter of Bayesian phylogenetic hierarchical linear models of intestinal length, diameter, and surface area for 122 species of coral reef fishes with a minimum sample size of five individuals per species. α = model intercept; $\beta_{SL\text{-}inter}$ = scaled species mean standard length slope; β_{TL} = scaled trophic level slope; $\beta_{SL\text{-}intra}$ = scaled species-mean-centred standard length slope; β_{EL} = scaled elongation slope; β_{ST} = stomach slope; β_{DU} = durophagy slope; $\beta_{ST:DU}$ = stomach X durophagy slope; σ_{phy} = random intercept standard deviation, phylogenetic relationship; σ_{sp} = random intercept standard deviation, species; $\sigma_{\beta sp}$ = random slope standard deviation, species; σ_{ϵ} = residual standard deviation; ν = degrees of freedom of student-t distribution.

parameter	Length mean	Length 2.5%	Length 97.5%	Diameter mean	Diameter 2.5%	Diameter 97.5%	Surface mean	Surface 2.5%	Surface 97.5%
α	5.77	5.17	6.37	1.56	1.31	1.81	8.44	7.65	9.22
$\beta_{SL\text{-}inter}$	0.40	0.32	0.47	0.40	0.34	0.45	0.77	0.67	0.88
β_{TL}	-0.18	-0.30	-0.08	-0.11	-0.18	-0.03	-0.27	-0.49	-0.09
$\beta_{SL\text{-}intra}$	0.13	0.12	0.15	0.11	0.10	0.13	0.25	0.22	0.28
β_{EL}	-0.33	-0.45	-0.21	-0.17	-0.24	-0.11	-0.50	-0.67	-0.34
β_{ST}	-0.33	-0.87	0.22	-0.30	-0.55	-0.06	-0.62	-1.36	0.11
β_{DU}	-0.26	-0.68	0.17	0.16	-0.06	0.39	-0.04	-0.61	0.52
$\beta_{ST:DU}$	0.05	-0.72	0.82	0.03	-0.32	0.38	0.02	-0.97	1.03
σ_{phy}	0.54	0.46	0.63	0.18	0.09	0.29	0.68	0.46	0.86
σ_{sp}	0.05	0.00	0.13	0.16	0.11	0.20	0.14	0.01	0.31
$\sigma_{\beta sp}$	0.05	0.04	0.07	0.05	0.02	0.07	0.10	0.06	0.13
σ_{ϵ}	0.16	0.14	0.17	0.19	0.18	0.21	0.28	0.26	0.30
ν	4.54	3.35	6.21	15.58	7.18	36.07	12.15	6.39	25.13

Table 4 Posterior mean and 95% credible interval for each parameter of Bayesian phylogenetic hierarchical linear models of intestinal length, diameter, and surface area for 69 species of coral reef fishes with a minimum sample size of eight individuals per species. α = model intercept; $\beta_{SL\text{-}inter}$ = scaled species mean standard length slope; β_{TL} = scaled trophic level slope; $\beta_{SL\text{-}intra}$ = scaled species-mean-centred standard length slope; β_{EL} = scaled elongation slope; β_{ST} = stomach slope; β_{DU} = durophagy slope; $\beta_{ST:DU}$ = stomach X durophagy slope; σ_{phy} = random intercept standard deviation, phylogenetic relationship; σ_{sp} = random

intercept standard deviation, species; $\sigma_{\beta sp}$ = random slope standard deviation, species; σ_{ϵ} = residual standard deviation; v = degrees of freedom of student-t distribution.

	Length			Diameter			Surface		
**parameter**	**mean**	**2.5%**	**97.5%**	**mean**	**2.5%**	**97.5%**	**mean**	**2.5%**	**97.5%**
α	5.49	4.73	6.26	1.63	1.30	1.99	8.34	7.39	9.29
$\beta_{SL\text{-}inter}$	0.30	0.18	0.42	0.36	0.27	0.45	0.64	0.47	0.81
β_{TL}	-0.22	-0.37	-0.09	-0.14	-0.22	-0.03	-0.33	-0.58	-0.11
$\beta_{SL\text{-}intra}$	0.14	0.11	0.16	0.11	0.09	0.13	0.25	0.21	0.28
β_{EL}	-0.35	-0.52	-0.19	-0.18	-0.28	-0.08	-0.53	-0.77	-0.31
β_{ST}	0.12	-0.64	0.89	-0.32	-0.69	0.02	-0.27	-1.23	0.67
β_{DU}	-0.02	-0.57	0.54	0.09	-0.23	0.39	0.02	-0.70	0.75
$\beta_{ST:DU}$	-0.48	-1.54	0.61	0.03	-0.46	0.53	-0.38	-1.72	0.99
σ_{phy}	0.53	0.42	0.66	0.17	0.03	0.35	0.66	0.40	0.86
σ_{sp}	0.06	0.00	0.17	0.16	0.07	0.23	0.14	0.01	0.33
$\sigma_{\beta sp}$	0.06	0.04	0.09	0.05	0.02	0.08	0.10	0.06	0.14
σ_{ϵ}	0.16	0.15	0.18	0.18	0.17	0.20	0.28	0.25	0.30
v	5.34	3.61	8.15	13.21	5.86	31.94	14.43	6.23	35.68

Fig. 2 Relationship between three intestinal traits and trophic level for: 142 species of coral reef fishes with a minimum sample size of three individuals per species (a, d, g); 122 species of coral reef fishes with a minimum sample size of three individuals per species (b, e, h); and 69 species of coral reef fishes with a minimum sample size of eight individuals per species (c, f, i). Thick, darkened lines represent the mean predicted fits of Bayesian phylogenetic hierarchical linear models after controlling for the remaining fixed and random effects. Categorical variables were set to their most common value (stomach = present, durophagy = non-durophagous). Thin lines represent 1,000 draws randomly chosen from the posterior fits and show model fit uncertainty. Model predictions are for natural-log intestinal traits, but are transformed here to show the fitted function on the original scale of the data. Raw data are displayed as marks along the x-axis.

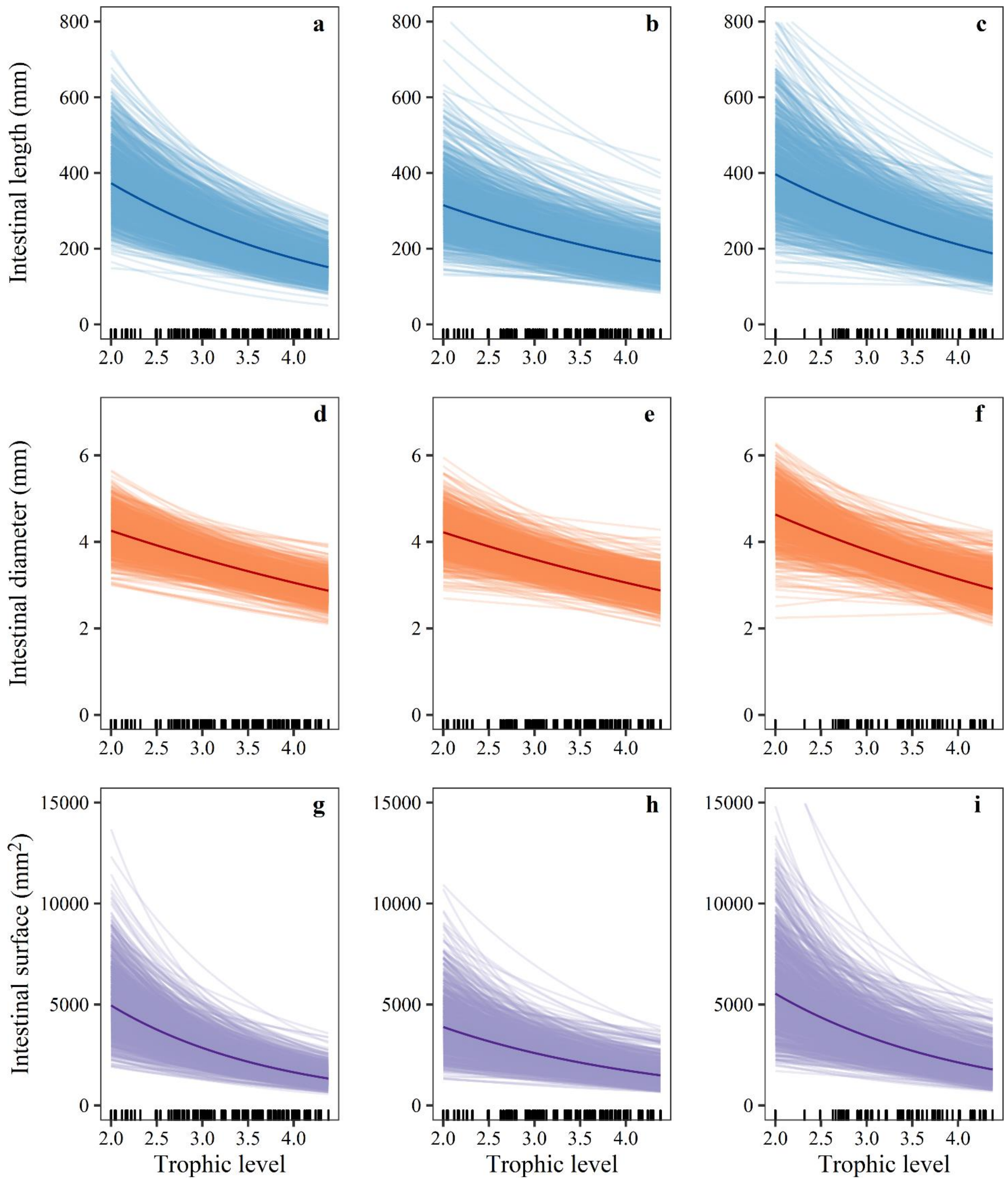

Fig. 3 Effects of stomach presence and durophagous diet on three intestinal traits for: 142 species of coral reef fishes with a minimum sample size of three individuals per species (a, b, c); 122 species of coral reef fishes with a minimum sample size of three individuals per species (d, e, f); and 69 species of coral reef fishes with a minimum sample size of eight individuals per species (g, h, i). Estimates are posterior medians (circles), 50% credible intervals (CIs; thick lines) and 95% CIs (thin lines) from Bayesian phylogenetic

hierarchical linear models after controlling for the remaining fixed and random effects. Posterior densities are also displayed (shaded regions).

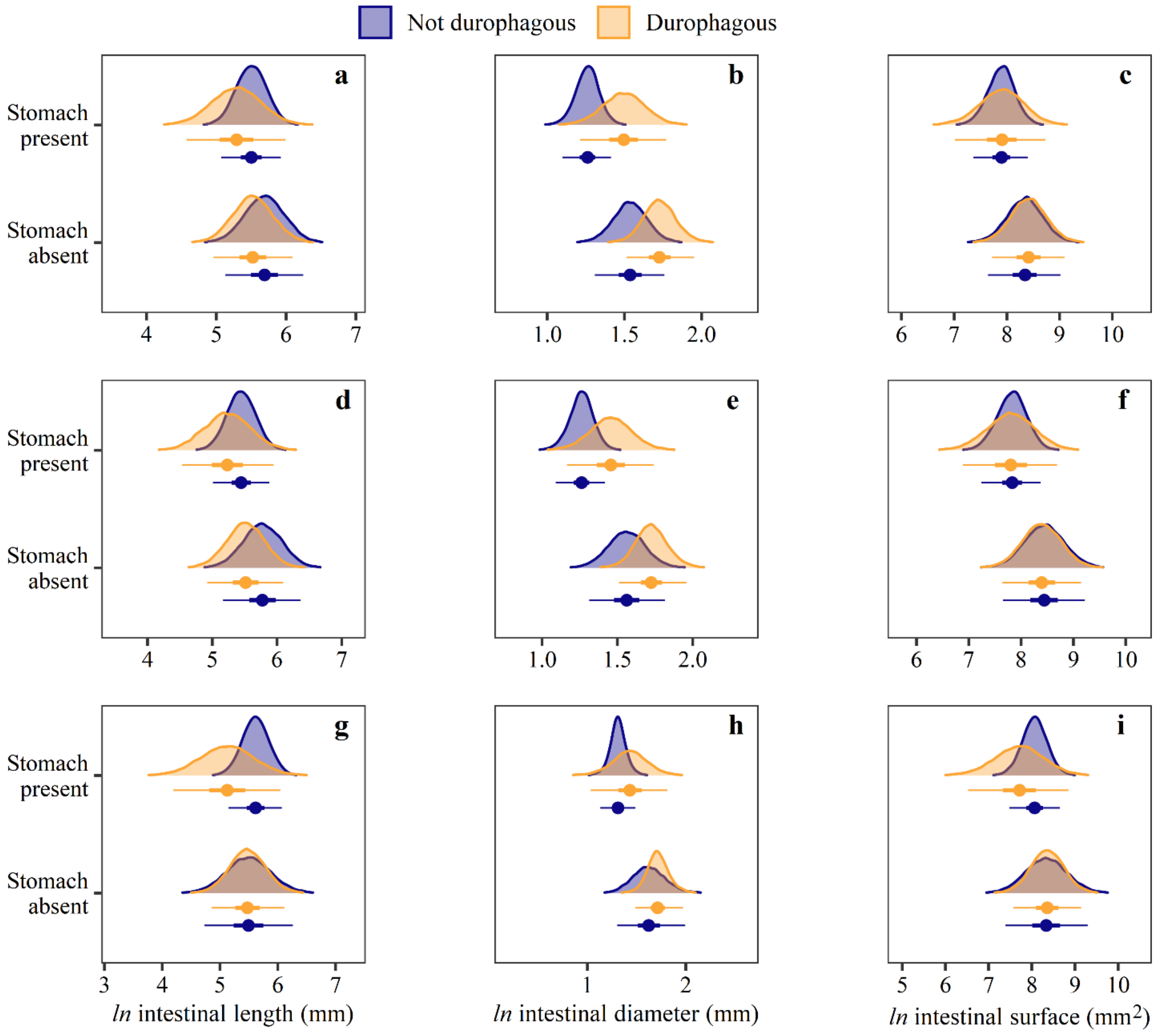

Table 5 Species-specific scaling parameters of three natural-log intestinal traits (intestinal length, diameter, and surface area) against natural-log fish standard length for 142 species of coral reef fishes. Estimates are posterior medians and 95% credible intervals (CIs) from Bayesian phylogenetic hierarchical linear models. Values in bold were selected based on a minimum number of ten sampled individuals, covering a length range of at least a quarter of the reported maximum length (retrieved from FishBase), and a posterior 95% CI above zero to provide reliable estimates of scaling parameters within the observed size range (see Fig. 5 in the main manuscript). The remaining estimates cannot be considered reliable. n = number of sampled individuals; TL = total length.

Family	Species	Length slope	Diameter slope	Surface slope	n	TL range (mm)
Acanthuridae	*Acanthurus achilles*	**1.17 (0.61, 1.68)**	**1.06 (0.57, 1.69)**	**2.50 (1.52, 3.43)**	12	122-222
Acanthuridae	*Acanthurus guttatus*	0.83 (0.17, 1.52)	0.67 (0.08, 1.20)	1.46 (0.34, 2.54)	9	161-198
Acanthuridae	*Acanthurus lineatus*	0.68 (0.11, 1.24)	0.69 (0.15, 1.18)	1.34 (0.38, 2.29)	9	160-252
Acanthuridae	*Acanthurus nigricans*	**0.93 (0.65, 1.24)**	**0.77 (0.47, 1.08)**	**1.74 (1.23, 2.26)**	13	82-184
Acanthuridae	*Acanthurus nigrofuscus*	0.71 (0.15, 1.28)	0.70 (0.21, 1.19)	1.39 (0.45, 2.33)	6	109-176
Acanthuridae	*Acanthurus nigros*	0.75 (0.03, 1.45)	0.59 (-0.02, 1.10)	1.14 (-0.01, 2.22)	4	119-156
Acanthuridae	*Acanthurus olivaceus*	0.80 (0.07, 1.54)	0.71 (0.11, 1.29)	1.49 (0.25, 2.70)	6	220-271
Acanthuridae	*Acanthurus pyroferus*	0.60 (-0.22, 1.33)	0.73 (0.13, 1.35)	1.30 (0.03, 2.53)	5	130-155
Acanthuridae	*Acanthurus thompsoni*	0.73 (-0.03, 1.46)	0.69 (0.08, 1.27)	1.32 (0.02, 2.55)	4	192-209
Acanthuridae	*Acanthurus triostegus*	0.76 (0.09, 1.43)	0.70 (0.13, 1.25)	1.47 (0.34, 2.59)	15	140-166
Acanthuridae	*Ctenochaetus flavicauda*	0.75 (0.02, 1.46)	0.67 (0.05, 1.23)	1.34 (0.12, 2.53)	6	108-139
Acanthuridae	*Ctenochaetus striatus*	0.91 (0.31, 1.53)	0.54 (-0.07, 1.02)	1.32 (0.31, 2.32)	5	120-215
Acanthuridae	*Naso brevirostris*	0.94 (0.38, 1.49)	0.74 (0.25, 1.24)	1.71 (0.77, 2.63)	6	224-343
Acanthuridae	*Naso lituratus*	**0.90 (0.33, 1.47)**	0.53 (-0.02, 0.98)	**1.22 (0.32, 2.09)**	17	181-356
Acanthuridae	*Naso unicornis*	1.07 (0.53, 1.65)	0.93 (0.48, 1.51)	2.16 (1.25, 3.14)	5	341-670
Acanthuridae	*Naso vlamingii*	0.46 (-0.04, 0.97)	0.54 (0.00, 0.97)	0.85 (-0.01, 1.69)	5	276-549
Acanthuridae	*Zebrasoma scopas*	**1.13 (0.91, 1.29)**	**0.84 (0.66, 1.02)**	**1.98 (1.69, 2.24)**	42	29-177
Acanthuridae	*Zebrasoma velifer*	0.89 (0.26, 1.54)	0.58 (-0.07, 1.08)	1.31 (0.23, 2.37)	9	196-259
Aulostomidae	*Aulostomus chinensis*	**1.24 (0.69, 1.78)**	**1.18 (0.69, 1.78)**	**2.71 (1.82, 3.62)**	11	365-623
Balistidae	*Balistapus undulatus*	**0.51 (0.11, 0.89)**	**0.79 (0.44, 1.16)**	**1.27 (0.63, 1.89)**	12	125-267
Balistidae	*Melichthys niger*	**1.12 (0.70, 1.57)**	**0.81 (0.44, 1.23)**	**1.88 (1.20, 2.61)**	14	125-258
Balistidae	*Melichthys vidua*	0.71 (0.08, 1.32)	0.79 (0.31, 1.34)	1.47 (0.52, 2.44)	12	176-225
Balistidae	*Odonus niger*	0.47 (-0.13, 1.04)	0.59 (0.06, 1.06)	0.94 (-0.04, 1.87)	14	158-277
Balistidae	*Rhinecanthus aculeatus*	0.46 (-0.15, 1.05)	0.71 (0.22, 1.20)	1.12 (0.20, 2.01)	9	145-222
Balistidae	*Rhinecanthus lunula*	0.79 (0.02, 1.54)	0.72 (0.12, 1.32)	1.51 (0.26, 2.78)	3	251-258
Balistidae	*Rhinecanthus rectangulus*	0.55 (0.04, 1.06)	0.71 (0.28, 1.15)	1.21 (0.42, 2.01)	9	117-195
Balistidae	*Sufflamen bursa*	0.53 (-0.05, 1.11)	**0.61 (0.09, 1.07)**	0.93 (-0.03, 1.85)	12	118-182
Blenniidae	*Exallias brevis*	0.95 (0.25, 1.69)	0.77 (0.21, 1.40)	1.81 (0.62, 3.05)	5	99-120
Bothidae	*Bothus mancus*	0.77 (0.02, 1.49)	0.68 (0.04, 1.26)	1.39 (0.12, 2.61)	3	366-405
Carangidae	*Caranx melampygus*	0.66 (0.11, 1.23)	0.65 (0.13, 1.13)	1.25 (0.33, 2.17)	5	349-545
Carangidae	*Scomberoides lysan*	0.78 (0.07, 1.47)	0.57 (-0.15, 1.11)	1.11 (-0.18, 2.31)	3	356-409
Chaetodontidae	*Chaetodon auriga*	0.70 (-0.10, 1.44)	0.62 (-0.01, 1.18)	1.21 (-0.10, 2.43)	9	145-205
Chaetodontidae	*Chaetodon bennetti*	0.77 (-0.01, 1.53)	0.71 (0.12, 1.31)	1.45 (0.15, 2.74)	3	151-168
Chaetodontidae	*Chaetodon citrinellus*	0.77 (0.02, 1.47)	0.77 (0.21, 1.39)	1.52 (0.32, 2.70)	7	96-113
Chaetodontidae	*Chaetodon ephippium*	0.91 (0.21, 1.62)	0.62 (-0.03, 1.15)	1.35 (0.11, 2.54)	8	157-194
Chaetodontidae	*Chaetodon lunula*	0.98 (0.40, 1.55)	0.57 (-0.02, 1.03)	1.24 (0.25, 2.19)	9	130-175
Chaetodontidae	*Chaetodon lunulatus*	1.14 (0.39, 1.89)	0.60 (0.08, 1.06)	1.84 (0.88, 2.79)	8	85-135
Chaetodontidae	*Chaetodon mertensii*	0.56 (-0.21, 1.28)	0.68 (0.08, 1.24)	1.08 (-0.23, 2.26)	5	103-129
Chaetodontidae	*Chaetodon ornatissimus*	**1.39 (1.11, 1.67)**	**0.56 (0.25, 0.85)**	**1.92 (1.45, 2.39)**	29	70-149
Chaetodontidae	*Chaetodon pelewensis*	0.81 (0.12, 1.49)	0.76 (0.19, 1.38)	1.61 (0.42, 2.82)	6	75-92

Family	Species	Length slope	Diameter slope	Surface slope	n	TL range (mm)
Chaetodontidae	*Chaetodon quadrimaculatus*	0.76 (0.08, 1.43)	0.68 (0.11, 1.21)	1.40 (0.32, 2.45)	8	97-129
Chaetodontidae	*Chaetodon reticulatus*	1.04 (0.60, 1.49)	0.79 (0.37, 1.24)	1.87 (1.14, 2.62)	32	86-130
Chaetodontidae	*Chaetodon trichrous*	0.95 (0.25, 1.69)	0.74 (0.16, 1.34)	1.72 (0.54, 2.94)	6	102-120
Chaetodontidae	*Chaetodon trifascialis*	0.97 (0.21, 1.77)	0.77 (0.20, 1.39)	1.91 (0.72, 3.17)	6	111-147
Chaetodontidae	*Chaetodon ulietensis*	0.68 (-0.08, 1.39)	0.67 (0.05, 1.22)	1.27 (0.02, 2.44)	8	134-158
Chaetodontidae	*Chaetodon unimaculatus*	0.42 (-0.22, 1.01)	0.58 (0.02, 1.06)	0.88 (-0.19, 1.86)	9	18-143
Chaetodontidae	*Chaetodon vagabundus*	0.53 (-0.25, 1.23)	0.69 (0.11, 1.25)	1.11 (-0.13, 2.21)	13	125-151
Chaetodontidae	*Forcipiger flavissimus*	0.80 (0.06, 1.50)	0.73 (0.14, 1.33)	1.52 (0.32, 2.70)	8	142-176
Chaetodontidae	*Forcipiger longirostris*	0.79 (0.08, 1.49)	0.74 (0.17, 1.33)	1.57 (0.38, 2.74)	7	170-222
Chaetodontidae	*Hemitaurichthys polylepis*	1.15 (0.56, 1.76)	0.88 (0.40, 1.50)	2.24 (1.23, 3.30)	7	97-140
Chaetodontidae	*Heniochus chrysostomus*	1.24 (0.53, 2.05)	0.70 (0.13, 1.25)	2.08 (1.00, 3.26)	8	110-142
Cirrhitidae	*Cirrhitus pinnulatus*	0.93 (0.39, 1.44)	0.72 (0.23, 1.20)	1.66 (0.74, 2.58)	10	126-190
Cirrhitidae	*Paracirrhites forsteri*	0.64 (0.06, 1.25)	0.67 (0.15, 1.16)	1.24 (0.27, 2.21)	9	124-183
Cirrhitidae	*Paracirrhites hemistictus*	0.86 (0.30, 1.43)	0.63 (0.09, 1.12)	1.45 (0.48, 2.40)	18	162-221
Diodontidae	*Diodon hystrix*	0.74 (0.06, 1.39)	0.81 (0.29, 1.44)	1.69 (0.59, 2.82)	5	300-404
Fistulariidae	*Fistularia commersonii*	**1.07 (0.60, 1.56)**	**0.95 (0.54, 1.45)**	**2.20 (1.41, 3.00)**	10	558-985
Gobiidae	*Valenciennea strigata*	1.14 (0.48, 1.81)	0.61 (0.04, 1.09)	1.67 (0.67, 2.66)	6	120-167
Hemiramphidae	*Hemiramphus depauperatus*	0.87 (0.11, 1.64)	0.70 (0.07, 1.29)	1.55 (0.23, 2.82)	3	230-271
Holocentridae	*Myripristis berndti*	**0.87 (0.39, 1.33)**	0.40 (-0.07, 0.79)	**0.96 (0.20, 1.72)**	16	120-260
Holocentridae	*Myripristis kuntee*	1.00 (0.35, 1.67)	0.49 (-0.20, 0.99)	1.10 (-0.01, 2.14)	7	139-178
Holocentridae	*Myripristis violacea*	0.99 (0.29, 1.71)	0.79 (0.25, 1.39)	1.85 (0.75, 3.01)	9	170-213
Holocentridae	*Neoniphon sammara*	**0.71 (0.07, 1.35)**	**0.58 (0.05, 1.03)**	**1.75 (0.91, 2.58)**	12	141-222
Holocentridae	*Sargocentron caudimaculatum*	0.95 (0.34, 1.56)	0.82 (0.32, 1.42)	1.93 (0.89, 3.01)	7	142-192
Holocentridae	*Sargocentron spiniferum*	0.91 (0.28, 1.55)	0.89 (0.39, 1.53)	1.94 (0.92, 3.00)	10	212-285
Holocentridae	*Sargocentron tiere*	**0.86 (0.17, 1.56)**	**0.67 (0.07, 1.22)**	**1.54 (0.37, 2.70)**	11	190-312
Kuhliidae	*Kuhlia mugil*	0.90 (0.15, 1.68)	0.70 (0.08, 1.31)	1.60 (0.36, 2.86)	3	251-282
Kyphosidae	*Kyphosus cinerascens*	0.84 (0.08, 1.60)	0.73 (0.13, 1.35)	1.59 (0.36, 2.87)	3	365-395
Kyphosidae	*Kyphosus vaigiensis*	0.83 (0.26, 1.39)	0.71 (0.20, 1.24)	1.54 (0.58, 2.51)	4	266-425
Labridae	*Calotomus carolinus*	0.67 (0.16, 1.17)	0.52 (-0.02, 0.95)	1.04 (0.17, 1.89)	9	261-410
Labridae	*Cheilinus chlorourus*	0.51 (-0.08, 1.08)	0.69 (0.18, 1.19)	1.15 (0.19, 2.09)	9	150-241
Labridae	*Cheilinus trilobatus*	1.18 (0.65, 1.70)	1.00 (0.54, 1.59)	2.39 (1.48, 3.29)	7	131-247
Labridae	*Cheilio inermis*	0.89 (0.17, 1.62)	0.81 (0.26, 1.47)	1.85 (0.65, 3.15)	4	322-391
Labridae	*Chlorurus microrhinos*	0.80 (0.06, 1.53)	0.69 (0.07, 1.28)	1.45 (0.20, 2.68)	4	360-390
Labridae	*Chlorurus spilurus*	0.58 (-0.12, 1.23)	0.90 (0.40, 1.56)	1.61 (0.51, 2.71)	6	187-475
Labridae	*Coris aygula*	1.00 (0.59, 1.40)	0.62 (0.22, 0.99)	1.59 (0.93, 2.23)	9	197-435
Labridae	*Coris gaimard*	1.01 (0.44, 1.52)	0.76 (0.31, 1.22)	1.76 (0.94, 2.58)	7	149-287
Labridae	*Epibulus insidiator*	0.56 (-0.08, 1.18)	0.66 (0.18, 1.11)	1.20 (0.36, 2.04)	16	170-300
Labridae	*Gomphosus varius*	0.93 (0.36, 1.50)	0.56 (-0.03, 1.05)	1.31 (0.33, 2.26)	7	183-280
Labridae	*Halichoeres hortulanus*	0.91 (0.40, 1.42)	0.91 (0.46, 1.48)	2.02 (1.14, 2.94)	9	164-261

Family	Species	Length slope	Diameter slope	Surface slope	n	TL range (mm)
Labridae	*Halichoeres trimaculatus*	0.78 (0.17, 1.39)	0.82 (0.31, 1.42)	1.75 (0.68, 2.84)	5	117-166
Labridae	*Hemigymnus fasciatus*	0.90 (0.39, 1.39)	0.70 (0.22, 1.17)	1.55 (0.73, 2.38)	9	145-229
Labridae	*Novaculichthys taeniourus*	0.72 (0.10, 1.32)	0.65 (0.08, 1.16)	1.28 (0.24, 2.30)	6	235-299
Labridae	*Oxycheilinus unifasciatus*	0.66 (-0.02, 1.33)	0.78 (0.25, 1.37)	1.53 (0.44, 2.63)	4	190-250
Labridae	*Scarus altipinnis*	0.70 (0.06, 1.34)	0.68 (0.11, 1.22)	1.36 (0.22, 2.45)	6	297-399
Labridae	*Scarus forsteni*	0.74 (0.16, 1.32)	0.66 (0.13, 1.13)	1.39 (0.40, 2.36)	9	235-382
Labridae	*Scarus frenatus*	0.05 (-0.53, 0.73)	0.66 (0.22, 1.07)	0.51 (-0.28, 1.29)	4	222-390
Labridae	*Scarus globiceps*	0.60 (-0.14, 1.27)	0.71 (0.19, 1.22)	0.87 (-0.16, 1.88)	7	196-261
Labridae	*Scarus oviceps*	0.85 (0.26, 1.43)	0.66 (0.12, 1.15)	1.47 (0.49, 2.44)	7	215-298
Labridae	*Scarus psittacus*	0.62 (0.04, 1.23)	0.46 (-0.10, 0.90)	1.10 (0.16, 2.00)	9	187-315
Labridae	*Scarus rubroviolaceus*	0.87 (0.20, 1.56)	0.96 (0.45, 1.64)	1.94 (0.82, 3.19)	5	255-454
Labridae	*Scarus schlegeli*	0.98 (0.40, 1.55)	0.83 (0.35, 1.39)	1.97 (1.02, 2.95)	7	193-301
Labridae	*Thalassoma hardwicke*	0.81 (0.29, 1.35)	0.65 (0.12, 1.11)	1.41 (0.49, 2.31)	7	119-202
Labridae	*Thalassoma purpureum*	0.91 (0.23, 1.58)	0.56 (-0.11, 1.08)	1.21 (0.05, 2.32)	5	230-287
Labridae	*Thalassoma trilobatum*	**0.67 (0.10, 1.22)**	**0.60 (0.07, 1.05)**	**1.11 (0.26, 1.92)**	10	166-284
Lethrinidae	*Gnathodentex aureolineatus*	0.77 (0.06, 1.46)	0.58 (-0.11, 1.11)	1.15 (-0.13, 2.33)	7	214-311
Lethrinidae	*Monotaxis grandoculis*	0.94 (0.31, 1.65)	0.55 (0.12, 0.92)	1.79 (0.92, 2.60)	7	158-365
Lutjanidae	*Aphareus furca*	0.84 (0.32, 1.36)	0.90 (0.46, 1.44)	1.81 (0.96, 2.67)	9	239-366
Lutjanidae	*Lutjanus fulvus*	1.31 (0.80, 1.80)	0.81 (0.39, 1.29)	2.19 (1.38, 3.02)	11	173-265
Lutjanidae	*Lutjanus kasmira*	0.89 (0.19, 1.60)	0.69 (0.09, 1.24)	1.54 (0.38, 2.68)	9	209-265
Lutjanidae	*Lutjanus monostigma*	0.88 (0.34, 1.39)	0.82 (0.35, 1.34)	1.77 (0.80, 2.69)	5	263-572
Monacanthidae	*Aluterus scriptus*	0.68 (0.17, 1.18)	0.81 (0.35, 1.31)	1.60 (0.74, 2.46)	4	328-616
Monacanthidae	*Amanses scopas*	0.47 (-0.08, 1.04)	0.67 (0.16, 1.14)	1.08 (0.18, 1.95)	9	104-168
Monacanthidae	*Cantherhines sandwichiensis*	0.45 (-0.21, 1.11)	**0.98 (0.50, 1.62)**	**1.60 (0.65, 2.55)**	10	115-172
Mugilidae	*Ellochelon vaigiensis*	0.72 (-0.02, 1.44)	0.67 (0.04, 1.24)	1.32 (0.06, 2.52)	4	340-398
Mullidae	*Mulloidichthys flavolineatus*	1.16 (0.63, 1.70)	0.69 (0.20, 1.18)	1.81 (0.93, 2.73)	9	194-319
Mullidae	*Mulloidichthys vanicolensis*	1.07 (0.49, 1.62)	0.92 (0.44, 1.51)	2.17 (1.19, 3.14)	5	187-297
Mullidae	*Parupeneus barberinus*	0.76 (0.35, 1.19)	0.81 (0.41, 1.22)	1.62 (0.93, 2.32)	4	200-440
Mullidae	*Parupeneus insularis*	**0.84 (0.46, 1.22)**	**0.69 (0.30, 1.07)**	**1.51 (0.86, 2.18)**	11	161-365
Mullidae	*Parupeneus multifasciatus*	**0.95 (0.52, 1.37)**	**0.93 (0.51, 1.42)**	**2.01 (1.23, 2.77)**	11	139-298
Ostraciidae	*Ostracion cubicus*	0.98 (0.25, 1.74)	0.67 (0.04, 1.23)	1.61 (0.41, 2.82)	3	244-298
Ostraciidae	*Ostracion meleagris*	0.59 (-0.01, 1.22)	0.66 (0.12, 1.15)	1.17 (0.22, 2.15)	7	90-121
Pempheridae	*Pempheris oualensis*	0.65 (-0.09, 1.34)	0.73 (0.15, 1.32)	1.37 (0.16, 2.53)	7	192-216
Pinguipedidae	*Parapercis millepunctata*	0.96 (0.31, 1.63)	0.80 (0.27, 1.42)	1.87 (0.76, 3.02)	7	114-145
Pomacanthidae	*Centropyge flavissima*	1.21 (0.76, 1.66)	0.76 (0.36, 1.19)	2.03 (1.31, 2.75)	8	55-101
Pomacanthidae	*Centropyge loriculus*	1.01 (0.28, 1.77)	0.80 (0.24, 1.44)	1.94 (0.80, 3.18)	3	50-64
Pomacanthidae	*Pomacanthus imperator*	0.93 (0.30, 1.54)	0.94 (0.47, 1.56)	1.99 (0.97, 3.02)	7	197-281
Pomacanthidae	*Pygoplites diacanthus*	**1.33 (0.70, 2.03)**	**0.86 (0.39, 1.42)**	**2.36 (1.37, 3.38)**	10	103-221

Family	Species	Length slope	Diameter slope	Surface slope	n	TL range (mm)
Pomacentridae	*Abudefduf septemfasciatus*	0.87 (0.18, 1.56)	0.73 (0.16, 1.29)	1.58 (0.46, 2.73)	11	161-194
Pomacentridae	*Abudefduf sexfasciatus*	0.93 (0.31, 1.57)	0.88 (0.38, 1.53)	2.07 (1.03, 3.19)	9	129-168
Pomacentridae	*Abudefduf sordidus*	0.69 (0.04, 1.32)	0.56 (-0.05, 1.04)	1.15 (0.09, 2.13)	6	147-228
Pomacentridae	*Chromis atripectoralis*	0.94 (0.24, 1.66)	0.72 (0.13, 1.30)	1.66 (0.52, 2.83)	5	54-68
Pomacentridae	*Chromis xanthura*	**0.61 (0.15, 1.08)**	**0.77 (0.36, 1.22)**	**1.46 (0.68, 2.24)**	24	111-158
Pomacentridae	*Dascyllus aruanus*	1.40 (0.92, 1.88)	0.71 (0.30, 1.11)	2.17 (1.46, 2.90)	7	29-54
Pomacentridae	*Dascyllus flavicaudus*	0.71 (0.20, 1.23)	0.50 (-0.03, 0.92)	0.96 (0.10, 1.79)	14	75-110
Pomacentridae	*Dascyllus trimaculatus*	0.74 (-0.03, 1.47)	0.65 (0.01, 1.22)	1.27 (0.00, 2.46)	8	120-135
Pomacentridae	*Plectroglyphidodon johnstonianus*	0.86 (0.14, 1.59)	0.79 (0.23, 1.42)	1.74 (0.58, 2.95)	6	59-76
Pomacentridae	*Plectroglyphidodon lacrymatus*	1.19 (0.60, 1.80)	0.71 (0.22, 1.20)	1.98 (1.07, 2.94)	6	50-91
Pomacentridae	*Stegastes fasciolatus*	0.78 (0.06, 1.47)	0.57 (-0.09, 1.11)	1.19 (-0.05, 2.33)	9	79-96
Pomacentridae	*Stegastes nigricans*	0.76 (0.01, 1.48)	0.76 (0.16, 1.38)	1.59 (0.35, 2.84)	10	120-141
Scorpaenidae	*Pterois radiata*	0.62 (0.14, 1.10)	0.62 (0.20, 1.01)	1.14 (0.41, 1.85)	9	117-197
Serranidae	*Cephalopholis argus*	0.85 (0.35, 1.33)	0.75 (0.32, 1.21)	1.60 (0.80, 2.39)	17	180-310
Serranidae	*Cephalopholis urodeta*	**0.74 (0.21, 1.38)**	**0.49 (0.01, 0.90)**	**1.03 (0.27, 1.85)**	14	105-191
Serranidae	*Epinephelus fasciatus*	0.62 (-0.03, 1.24)	0.65 (0.10, 1.16)	1.19 (0.16, 2.20)	6	190-288
Serranidae	*Epinephelus hexagonatus*	0.97 (0.36, 1.58)	0.76 (0.24, 1.32)	1.88 (0.86, 2.91)	7	152-225
Serranidae	*Epinephelus merra*	0.85 (0.25, 1.44)	0.88 (0.39, 1.49)	1.89 (0.89, 2.94)	9	165-230
Serranidae	*Pseudanthias pascalus*	0.67 (0.13, 1.22)	0.80 (0.30, 1.34)	1.56 (0.61, 2.48)	7	89-152
Serranidae	*Variola louti*	0.76 (0.24, 1.33)	0.61 (0.06, 1.08)	1.26 (0.34, 2.20)	7	304-420
Siganidae	*Siganus spinus*	0.67 (0.05, 1.26)	0.80 (0.28, 1.38)	1.56 (0.52, 2.59)	5	192-223
Tetraodontidae	*Arothron meleagris*	0.63 (0.16, 1.15)	0.88 (0.47, 1.35)	1.60 (0.89, 2.34)	9	141-269
Tetraodontidae	*Canthigaster bennetti*	0.72 (-0.08, 1.47)	0.75 (0.16, 1.37)	1.41 (0.16, 2.62)	3	79-94
Tetraodontidae	*Canthigaster solandri*	0.66 (-0.02, 1.32)	0.72 (0.16, 1.27)	1.33 (0.24, 2.40)	6	70-97
Zanclidae	*Zanclus cornutus*	0.77 (0.13, 1.41)	0.77 (0.24, 1.36)	1.61 (0.52, 2.70)	11	131-171

Fig. 4 Effect of phylogeny on three intestinal traits for 142 species of coral reef fishes. Estimates are posterior medians (circles), 50% credible intervals (CIs; thick lines) and 95% CIs (thin lines) from Bayesian phylogenetic hierarchical linear models, and represent deviations from the global intercept on natural-log scale.

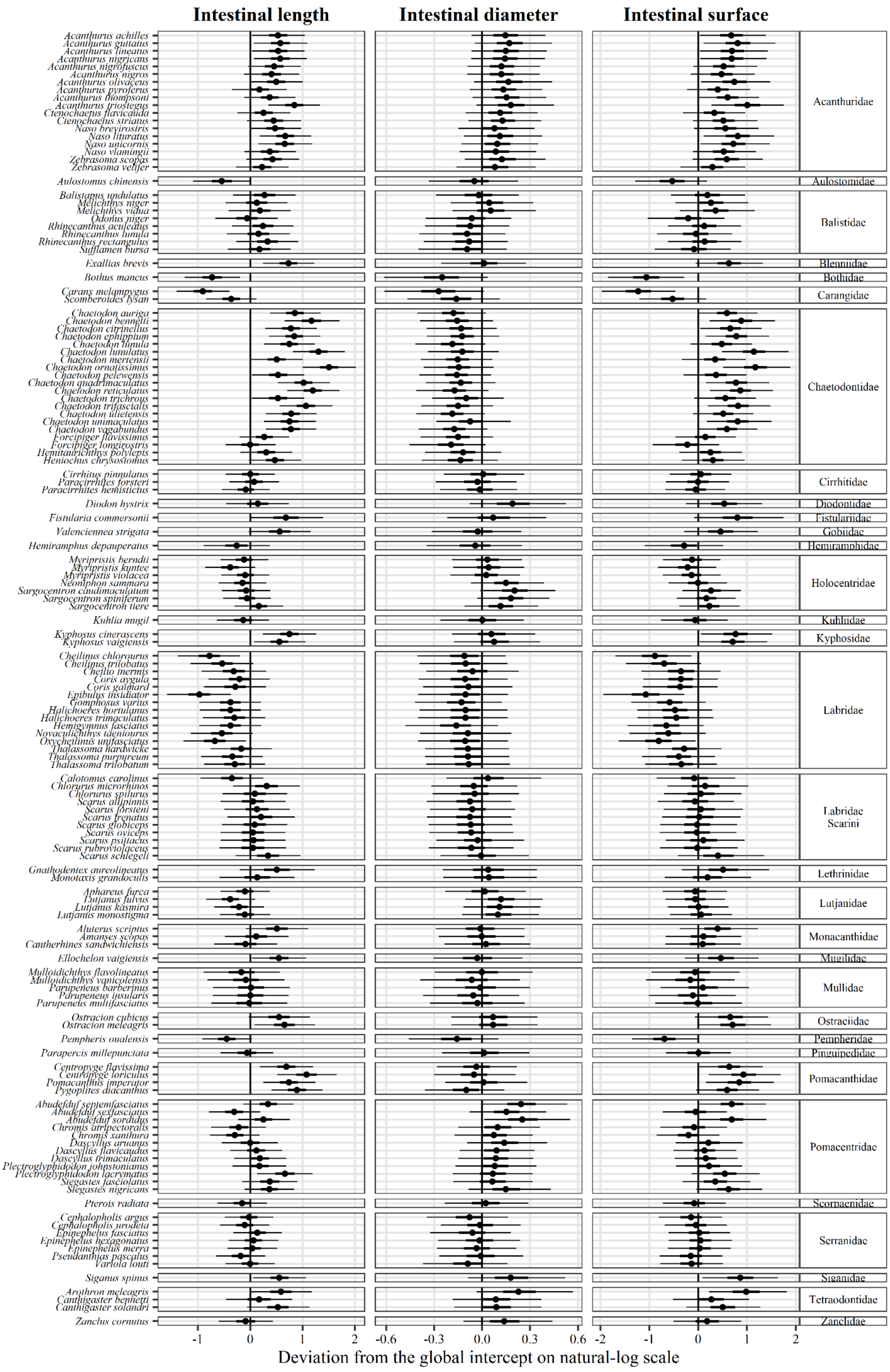

Intestinal length
Intestinal diameter
Intestinal surface
Acanthurus achilles
Acanthurus guttatus
Acanthurus lineatus
Acanthurus nigricans
Acanthurus nigrofuscus
Acanthurus nigros
Acanthurus olivaceus
Acanthurus pyroferus
Acanthurus thompsoni
Acanthurus triostegus
Ctenochaetus flavicauda
Ctenochaetus striatus
Naso brevirostris
Naso lituratus
Naso unicornis
Naso vlamingii
Zebrasoma scopas
Zebrasoma velifer
Acanthuridae
Aulostomus chinensis
Aulostomidae
Balistapus undulatus
Melichthys niger
Melichthys vidua
Odonus niger
Rhinecanthus aculeatus
Rhinecanthus lunula
Rhinecanthus rectangulus
Sufflamen bursa
Balistidae
Exallias brevis
Blenniidae
Bothus mancus
Bothidae
Caranx melampygus
Scomberoides lysan
Carangidae
Chaetodon auriga
Chaetodon bennetti
Chaetodon citrinellus
Chaetodon ephippium
Chaetodon lunula
Chaetodon lunulatus
Chaetodon mertensii
Chaetodon ornatissimus
Chaetodon pelewensis
Chaetodon quadrimaculatus
Chaetodon reticulatus
Chaetodon trichrous
Chaetodon trifascialis
Chaetodon ulietensis
Chaetodon unimaculatus
Chaetodon vagabundus
Forcipiger flavissimus
Forcipiger longirostris
Hemitaurichthys polylepis
Heniochus chrysostomus
Chaetodontidae
Cirrhitus pinnulatus
Paracirrhites forsteri
Paracirrhites hemisticus
Cirrhitidae
Diodon hystrix
Diodontidae
Fistularia commersonii
Fistulariidae
Valenciennea strigata
Gobiidae
Hemiramphus depauperatus
Hemiramphidae
Myripristis berndti
Myripristis kuntee
Myripristis violacea
Neoniphon sammara
Sargocentron caudimaculatum
Sargocentron spiniferum
Sargocentron tiere
Holocentridae
Kuhlia mugil
Kuhliidae
Kyphosus cinerascens
Kyphosus vaigiensis
Kyphosidae
Cheilinus chlorourus
Cheilinus trilobatus
Cheilio inermis
Coris aygula
Coris gaimard
Epibulus insidiator
Gomphosus varius
Halichoeres hortulanus
Halichoeres trimaculatus
Hemigymnus fasciatus
Novaculichthys taeniourus
Oxycheilinus unifasciatus
Thalassoma hardwicke
Thalassoma purpureum
Thalassoma trilobatum
Labridae
Calotomus carolinus
Chlorurus microrhinos
Chlorurus spilurus
Scarus altipinnis
Scarus forsteni
Scarus frenatus
Scarus globiceps
Scarus oviceps
Scarus psittacus
Scarus rubroviolaceus
Scarus schlegeli
Labridae
Scarini
Gnathodentex aureolineatus
Monotaxis grandoculis
Lethrinidae
Aphareus furca
Lutjanus fulvus
Lutjanus kasmira
Lutjanus monostigma
Lutjanidae
Aluterus scriptus
Amanses scopas
Cantherhines sandwichiensis
Monacanthidae
Ellochelon vaigiensis
Mugilidae
Mulloidichthys flavolineatus
Mulloidichthys vanicolensis
Parupeneus barberinus
Parupeneus insularis
Parupeneus multifasciatus
Mullidae
Ostracion cubicus
Ostracion meleagris
Ostraciidae
Pempheris oualensis
Pempheridae
Parapercis millepunctata
Pinguipedidae
Centropyge flavissima
Centropyge loriculus
Pomacanthus imperator
Pygoplites diacanthus
Pomacanthidae
Abudefduf septemfasciatus
Abudefduf sexfasciatus
Abudefduf sordidus
Chromis atripectoralis
Chromis xanthura
Dascyllus aruanus
Dascyllus flavicaudus
Dascyllus trimaculatus
Plectroglyphidodon johnstonianus
Plectroglyphidodon lacrymatus
Stegastes fasciolatus
Stegastes nigricans
Pomacentridae
Pterois radiata
Scorpaenidae
Cephalopholis argus
Cephalopholis urodeta
Epinephelus fasciatus
Epinephelus hexagonatus
Epinephelus merra
Pseudanthias pascalus
Variola louti
Serranidae
Siganus spinus
Siganidae
Arothron meleagris
Canthigaster bennetti
Canthigaster solandri
Tetraodontidae
Zanclus cornutus
Zanclidae
-1
0
1
2
-0.6
-0.3
0.0
0.3
0.6
-2
-1
0
1
2
Deviation from the global intercept on natural-log scale
